# USP48 and A20 synergistically promote cell survival in *Helicobacter pylori* infection

**DOI:** 10.1007/s00018-022-04489-7

**Published:** 2022-08-01

**Authors:** Phatcharida Jantaree, Supattra Chaithongyot, Olga Sokolova, Michael Naumann

**Affiliations:** Institute of Experimental Internal Medicine, Medical Faculty, Otto Von Guericke University, Leipziger Str. 44, 39120 Magdeburg, Germany

**Keywords:** Apoptotic cell death, COP9 signalosome (CSN), Deubiquitinylase (DUB), Nedd8, RelA

## Abstract

The human pathogen *Helicobacter pylori* represents a risk factor for the development of gastric diseases including cancer. The *H. pylori*-induced transcription factor nuclear factor kappa-light-chain-enhancer of activated B cells (NF-κB) is involved in the pro-inflammatory response and cell survival in the gastric mucosa, and represents a trailblazer of gastric pathophysiology. Termination of nuclear NF-κB heterodimer RelA/p50 activity is regulated by the ubiquitin-RING-ligase complex elongin-cullin-suppressor of cytokine signalling 1 (ECS^SOCS1^), which leads to K48-ubiquitinylation and degradation of RelA. We found that deubiquitinylase (DUB) ubiquitin specific protease 48 (USP48), which interacts with the COP9 signalosome (CSN) subunit CSN1, stabilises RelA by deubiquitinylation and thereby promotes the transcriptional activity of RelA to prolong de novo synthesis of DUB A20 in *H. pylori* infection. An important role of A20 is the suppression of caspase-8 activity and apoptotic cell death. USP48 thus enhances the activity of A20 to reduce apoptotic cell death in cells infected with *H. pylori*. Our results, therefore, define a synergistic mechanism by which USP48 and A20 regulate RelA and apoptotic cell death in *H. pylori* infection.

**Supplementary Information:**

The online version contains supplementary material available at 10.1007/s00018-022-04489-7.

## Introduction

Infection of *H. pylori*, a Gram-negative microaerophilic spiral bacterium, has been observed in nearly half of the world population [1]. *H. pylori* specifically colonises the gastric mucosa and is a risk factor for chronic gastritis, initiating precancerous lesions, which may then progress to metaplasia [2]. At the cellular level, recent studies revealed that *H. pylori* ADP-glycero-*β*-d-manno-heptose (ADP heptose), as the activator of the proteins *α*-kinase 1 (ALPK1) and subsequently tumour necrosis factor receptor-associated factors (TRAF)-interacting protein with forkhead-associated domain (TIFA), induces classical and alternative NF-κB in gastric epithelial cells [3, 4]. Activation of NF-κB is strictly dependent on the type IV secretion system (T4SS) and independent of the cytotoxin A gene (CagA) [5].

Excessive and deregulated NF-κB activation can cause massive damage to host tissues and contributes to the pathogenic processes of various inflammatory diseases [6]. A crucial mechanism for the timely termination of NF-κB activity is controlled by the ubiquitin–proteasome system (UPS)-dependent degradation of RelA in the nucleus [7]. Ubiquitinylation is regulated by an enzymatic cascade of E1 (activating enzymes), E2 (conjugating enzymes) and E3 (ligases) [8]. In TNF stimulation, the E3 cullin-RING-ubiquitin ligase (CRL) ECS^SOCS1^ ubiquitinylates nuclear RelA [9], which is, in subnuclear structures (promyelocytic leukaemia protein nuclear bodies (PML bodies)), degraded by the 26S proteasome [10].

Ubiquitin ligation by CRLs is controlled by the protein complex CSN, which removes the CRL activating Ub-like molecule neural precursor cell expressed developmentally downregulated gene 8 (NEDD8) from cullin subunits [11]. The CSN composes of eight subunits, including six proteasome-COP9-initiation factor 3 (PCI) domain subunits and two Mov34-and-Pad1p N-terminal (MPN) domain subunits (CSN5, CSN6) situated on top of the helical bundle [12]. Of note, CSN5 possess the JAB1/MPN/Mov34 metalloenzyme (JAMM) motif of DUB families and is responsible for deneddylase activity [13]. Moreover, the CSN is associated with the DUBs USP15 [14], USP48 [15], CYLD [16] and STAMBPL1 [17], which control the activity of a variety of target molecules [11]. DUBs reverse ubiquitinylation by hydrolysis of the isopeptide bond between ubiquitin and the targeted substrate or the peptide between individual ubiquitins to facilitate complete removal or modification of the ubiquitin signal [18].

CSN-associated USP48 removes K48-linked polyubiquitin chains from nuclear RelA and promotes NF-κB transcriptional activity of target genes, including tumour necrosis factor alpha-induced protein 3 (*TNFAIP3)*, which encodes for the de novo synthesised DUB A20 upon TNF stimulation [15]. A20 is a zinc finger protein possessing an ovarian tumour (OTU) domain that serves via its DUB activity as a crucial negative regulator in NF-κB signalling. A20 deficient cells are hypersensitive to NF-κB activation by multiple stimuli, including TNF, interleukin 1*β* (IL-1*β*), toll like receptor (TLR) ligands and retinoic-acid-inducible protein 1 (RIG-1)-like receptor (RLR) ligands [19]. A20 interferes with the components of the NF-κB signalling cascade and terminates NF-κB activation, e.g. by interacting with the linear polyubiquitin chain of NF-κB essential modulator (NEMO), thereby preventing activation of the IκB kinases (IKKs) [20]. In addition, A20 enzymatically counteracts cullin3-mediated K63-linked ubiquitinylation of procaspase-8, suppressing caspase-8 activity and apoptotic cell death [21, 22]. On the other hand, A20 suppresses alternative NF-κB activity and thus the anti-apoptotic genes baculoviral IAP repeat containing 2 (*BIRC2)*, *BIRC3* and B-cell lymphoma 2-related protein A1 (*BCL2A1)*, promoting apoptotic cell death [23].

## Material and methods

### Cell culture and *H. pylori* infection

AGS (ATCC, CRL-1739) and NCI-N87 (ATCC, CRL-5822) cells were cultured in RPMI 1640 medium (Gibco^®^/Life Technologies) supplemented with 10% foetal calf serum (FCS) (Biochrom) at 37 °C, 5% CO_2_ in a humidified atmosphere. The culture medium was changed to fresh RPMI 1640 medium supplemented with 0.2% FCS overnight before infection with *H. pylori*.

*H. pylori* strain P1 [24] was grown on GC agar plates supplemented with 10% horse serum (Gibco^®^/Life Technologies), 5 μg/ml trimethoprim (Sigma-Aldrich), 1 μg/ml nystatin (Sigma-Aldrich) and 10 μg/ml vancomycin (Sigma-Aldrich) at 37 °C under microaerophilic conditions for 48 h before infection. For infection, *H. pylori* were prepared in phosphate-buffered saline (PBS), and the cells were infected at a multiplicity of infection (MOI) of 100.

### Transactivation assay

AGS cells were seeded in 24-well plate at a density of 60,000 cells/well. Firefly luciferase plasmid containing five copies of an NF-κB response element (Promega) was mixed with *Renilla* Luciferase plasmid at a ratio of 50:1 and transfected using Attractene^®^ transfection reagent (Qiagen) for 48 h. After 3.5 h of *H. pylori* infection or IL-1β (10 ng/ml) stimulation, luciferase activity was estimated in cell lysates using a Dual-Luciferase Reporter Assay System (Promega) with a Lumat LB 9507 luminometer (Berthold Technologies). The inducible firefly luciferase activity was normalized relative to *Renilla* luciferase activity, and fold changes in stimulated samples were calculated compared to non-stimulated cells.

### Transfection of siRNA and protein

Cells were seeded at 0.4 × 10^6^ per 60-mm or 1.4 × 10^6^ per 100-mm culture dish one day before transfection. Transfection of siRNA was performed using siLentFect™ (Bio-Rad, #1703362) following the manufacturer’s instructions. Briefly, the cell culture medium was changed to Opti-MEM prior to transfection. siRNA against Elongin B, USP48, CSN2, and A20 were prepared at a final concentration of 40 nM. The following siRNAs were used: USP48^si1^ (s38642, ambion®/Life Technologies), USP48^si5^ (s38644, ambion®/Life Technologies), A20^si9^ (SI05018601, Qiagen), CSN2^si^ (AM16708, Thermo Fisher Scientific), Elongin B^si^ 5’-UGACCAACUCUUGGAUGAU-3’ (Eurofins Genomics), Caspase-8^si^ (#SI02661946, Qiagen) and scrambled^si^ (#D-001810–10, Dharmacon). At 6 h after transfection, the medium was changed to fresh RPMI 1640 medium containing 10% FCS and the cells cultured for additional 42 h before infection. The transfection of His-tagged recombinant human USP48 protein (#E-614, Boston Biochem™) or recombinant human A20 protein (#80408, BPS Bioscience) was performed as follows: 1 μg recombinant USP48 protein was mixed with 2 μl Cas9 PLUS™ reagent (Thermo Fisher Scientific) in 125 μl Opti-MEM and incubated for 5 min at room temperature (RT). 4 μl CRISPRMAX transfection reagent (Thermo Fisher Scientific) was diluted in 125 μl Opti-MEM. The USP48 protein/Cas9 PLUS™ solution was combined with the CRISPRMAX transfection solution, followed by incubation at RT for 20 min. The cell culture medium was changed to fresh RPMI 1640 medium containing 10% FCS before adding dropwise the transfection solution to the cells in the dish and the cells cultured for additional 1 h before infection.

### Preparation of whole-cell lysates and subcellular fractionation

MG132 (20 μM, Tocris) and Leptomycin B (LMB, 10 ng/ml, Calbiochem) were added to the culture medium 30 min after *H. pylori* infection when required, as indicated. For whole-cell lysates, the cells were washed twice with ice-cold PBS and lysed in RIPA lysis buffer (50 mM Tris (pH 7.5), 150 mM NaCl, 2 mM EDTA, 10 mM K_2_HPO_4_, 10% glycerol, 1% Triton X-100, 0.05% SDS) supplemented with 1 mM Na_3_VO_4_, 1 mM Na_2_MoO_4_, 20 mM NaF, 10 mM Na_4_P_2_O_7_, 1 mM AEBSF, 20 mM Glycerol-2-phosphate, and 1 × EDTA-free protease inhibitor mix (PI) (cOmplete™, Mini, Roche). *N*-ethyl-maleimide (NEM) (7.5 mM, Fluka) and ortho-phenanthroline (OPT) (5 mM, Sigma-Aldrich) were added to the lysis buffer when preservation of ubiquitinylated proteins was required. Lysates were obtained after centrifugation (13,000*g*, 4 °C, 10 min).

Subcellular fractionation was performed as previously described [15, 25] with some modifications. The cells were washed twice with ice-cold PBS and gently scraped in pre-chilled buffer A (20 mM Tris (pH 7.9), 10 mM NaCl, 1.5 mM MgCl_2_, 10 mM K_2_HPO_4_, and 10% Glycerol) supplemented with 1 × PI, 1 mM Na_3_VO_4_, 1 mM Na_2_MoO_4_, 10 mM NaF, 0.5 mM AEBSF, 20 mM Glycerol-2-phosphate and 0.5 mM DTT. The cells were allowed to swell for 10 min on ice. Afterwards, 1 μl of 12.5% NP-40 was added per 100 μl cell suspension to burst the cytoplasmic membrane and incubated for 5 min on ice. Then, nuclei were separated from cytosolic supernatants by centrifugation (2000*g*, 4 °C, 10 min) and cytosolic fractions were cleared (13,000*g*, 4 °C, 10 min). Nuclear pellets (P1) were washed once with buffer A and then resuspended in buffer C (20 mM Tris (pH 7.9), 420 mM NaCl, 1.5 mM MgCl_2_, 0.2 mM EDTA, 10 mM K_2_HPO_4_, and 10% Glycerol), supplemented as described for buffer A, to extract soluble nuclear proteins (N1). The suspension was incubated for 30 min on ice with occasional vortexing. Afterwards, N1 fractions were cleared by centrifugation (13,000*g*, 4 °C, 10 min). The pellets (P2) with the subnuclear fractions were resuspended in buffer E (20 mM Tris (pH 7.9), 150 mM NaCl, 1.5 mM MgCl_2_, 5 mM CaCl_2_, 10% Glycerol and 2% SDS) supplemented as described for buffer A with additional Benzonase^®^ Nuclease (25 U/ml, Novagen), DTT omitted. After incubating the suspension for 30 min on ice, the N2 fractions were cleared by centrifugation (13,000*g*, 4 °C, 10 min). NEM (7.5 mM) and OPT (5 mM) were added to the fractionation buffers when preservation of ubiquitinylated protein was required. Protein concentration was determined using the Pierce™ BCA protein assay kit (Thermo Fisher Scientific), according to the manufacturer’s instructions.

### Immunoprecipitation and immunoblotting

For immunoprecipitation (IP) from cell lysates, equal amounts of protein were diluted with RIPA buffer supplemented with 1 mM Na_3_VO_4_, 1 mM Na_2_MoO_4_, 20 mM NaF, 10 mM Na_4_P_2_O_7_, 1 mM AEBSF, 20 mM Glycerol-2-phosphate, and 1 × PI. 1 μg protein-specific antibody was added per IP and incubated overnight on a permanent rotator (7 rpm) at 4 °C. Pre-washed Pierce™ protein A/G magnetic beads (#88802, Thermo Fisher Scientific) were added and additionally rotated for 2 h at 4 °C. The beads were washed three times with RIPA buffer and twice with PBS, then eluted in 2 × Laemmli sample buffer. For the analysis of ubiquitinylated proteins, the buffer was additionally supplemented with NEM (7.5 mM), OPT (5 mM). For the IP under denaturing conditions, the cells were lysed in the buffer containing 1% SDS and the IP was performed with 0.1% SDS.

For SDS–PAGE and immunoblotting, samples were heated at 95 °C for 10 min, separated in Tris–Glycine gels and transferred onto PVDF membranes (Millipore). The membranes were blocked with 5% skim milk in TBS containing 0.1% Tween 20 (TBS-T) at RT for 1 h and incubated with primary antibodies in either 5% BSA or 5% skim milk in TBS-T at 4 °C overnight. The membranes were washed thrice with TBS-T before incubating with appropriate HRP‐conjugated secondary antibody in 5% skim milk in TBS-T at RT for 1 h. The membranes were washed thrice with TBS-T and then developed using a chemiluminescent substrate (#WBKLS0500, Millipore). The band pattern was visualised using the ChemoCam Imager (Intas).

The following primary antibodies were used: A20 (sc-166692), C23 (sc-13057), CSN6 (sc-137153), Elongin B (sc-11447), Lamin B2 (sc-377379), RelA (sc-8008) and Ubquitin (sc-8017) were purchased from Santa Cruz Biotechnology; Caspase 3 (#9662), Caspase 8 (#9746), Cleaved Caspase 3 (#9661), IκBα (#4812), phospho-RelA (#3031) were purchased from Cell Signalling Technology; CSN2 (ab155774) and USP48 (ab72226) were purchased from Abcam; GAPDH (#MAB374) and Ubiquitin K63 linkage (05-1308)) were purchased from Millipore; CSN1 (BML-PW8285-0100, ENZO); CSN5 (GTX70207, GeneTex); FLAG (#F3165, Sigma-Aldrich).

### RNA isolation, reverse transcription and quantitative PCR

Total RNA was isolated from cultured cells using the Nucleospin^®^ RNA Plus kit (Macherey–Nagel), following the manufacturer’s protocol. RNA concentration was measured using the NanoDrop spectrophotometer. The isolated RNA was then reverse-transcribed into cDNA using RevertAid First Strand cDNA Synthesis Kit (Thermo Fisher Scientific). All steps were performed under nuclease-free conditions. The SensiMix^®^ Hi-ROX (Bioline) was used to analyse the gene transcripts. The quantitative PCR was performed using the StepOnePlus™ Real-Time PCR system (Applied Biosystem, Thermo Fisher Scientific). The comparative *C*_*T*_ method (*ΔΔC*_*T*_) was used to quantify relative changes in the target mRNA by normalisation on a reference gene (*GAPDH*). The following primer pairs were used: *NFKBIA* (5′-GCAGACTCCACTCCACTTG-3′ fw; 5′-CGTCCTCTGTGAACTCCG-3′ rev), *CXCL8* (5′-AGATGTCAGTGCATAAAGACA-3′ fw; 5′-TATGAATTCTCAGCCCTCTTCAAAAA-3′ rev), *TNFAIP3* (5′-CTGAAATCCGAGCTGTTCCAC-3′ fw; 5′-GAGATGAGTTGTGCCATGGTC-3′ rev), *USP48* (5′-GGCAGGTGGCGAAGCCCATT-3′ fw; 5′-CAGTTTCGTCTGCACACGCCG-3′ rev) and *GAPDH* (5′-CATCACCATCTTCCAGGAGC-3′ fw; 5′-CATACTTCTCATGGTTCACACC-3′ rev) were from Eurofins Genomics.

### In vitro translation and binding assay

The CSN proteins were in-vitro translated using the PURExpress^®^ In Vitro Protein Synthesis Kit (New England Biolabs), following the manufacturer’s protocol. Each translated protein was incubated with 0.5 μg recombinant human USP48 (E-614, Boston Biochem™) for 1 h at 37 °C followed by IP using an anti-USP48 antibody (ab72226, Abcam). IP buffer (20 mM Tris–HCl, 150 mM NaCl, 2 mM EDTA, 1% TritonX100, 0.1% SDS; pH 7.4) was supplemented with 2 mM Na_3_VO_4_, 20 mM NaF, 1 × PI. The IPs were analysed by SDS–PAGE and IB.

### In vitro DUB assay

RelA was immunoprecipitated from the total nuclear fraction of *H. pylori*-infected cells treated with MG132 using low-pH elution buffer (0.1 M glycine, pH 2.5). The eluted IP samples were immediately neutralised by 1 M Tris–HCl, pH 8.5 (1:10 of eluted volume).

Recombinant USP48 (0.5 μM) was pre-incubated for 5 min at 30 °C with continuous shaking in DUB assay buffer (50 mM HEPES (pH 7.5) with 100 mM NaCl) supplemented with 1 mM DTT and 1 mM AEBSF. Next, ubiquitinylated immunoprecipitated RelA was added and incubated in the presence or absence of NEM (10 mM) in a final reaction volume of 15 μl at 30 °C with continuous shaking. Reactions were stopped after 2 h incubation by addition of 5 μl 4 × Laemmli sample buffer and 2 μl β-ME and then boiled at 95 °C for 5 min. Samples were then separated by SDS-PAGE and analysed by IB.

### Apoptotic cell death analysis

The cells were trypsinised and stained with an Annexin V-FITC/PI Kit (MabTag GmbH), according to the manufacturer’s instructions. Apoptotic cell death was determined using a flow cytometer (CyFlow space). Data were processed using Flowing Software 2.

### Caspase 3/7 assay

After 24 h of siRNA transfection, cells were re-seeded into a 24-well plate at a density of 50,000 cells/well and allowed to adhere overnight. Transfection of recombinant human USP48 protein was performed as described before. The culture media were replaced by media containing *H. pylori* (MOI 100), Incucyte^®^ Caspase 3/7 Green Dye (staining of apoptotic cells, dilution 1:1000, Sartorius) and Incucyte^®^ NucLight Rapid Red Dye (staining of all cells, dilution 1:1000, Sartorius). The fluorescence signal was measured by the Incucyte^®^ S3 Live-Cell Analysis System (Sartorius), and image sets of at least four pictures from distinct regions per well were captured with phase contrast and fluorescence channels at a magnification of 20 × . The number of fluorescence-stained cells and fluorescence intensity were acquired using Incucyte^®^ S3 Live-Cell Analysis System integrated software, and presented as percentages of apoptotic cells or fluorescence intensity (A.U.).

### Caspase-Glo™ 8 assay

Luminescence assay for caspase-8 activity was performed using Caspase-Glo™ 8 Assay Kit (Promega). siRNA transfected cells were plated into a white-walled 96-well plate at a density of 10,000 cells/well. The cells were allowed to grow for 48 h. The culture media were changed to 100 μl media containing H. pylori (MOI 100) and incubated for 24 h. Prior to starting the assay, the Caspase-Glo^®^ 8 Reagent was prepared according to the manufacturer's instructions. Then, 100 μl of Caspase-Glo^®^ 8 Reagent was added to each well and gently mixed contents of the wells using a plate shaker at 300 rpm for 1 min. The plate was then incubated at room temperature for 1 h. After that, the luminescence of each sample was measured using a luminometer (SpectraMax M5, Molecular Devices), and caspase-8 activity (fold increase) was calculated.

### Statistical analysis

Quantitative data from at least 3 independent experiments were presented as mean ± SD (standard deviation). Statistical significance of data was analysed applying Student’s T test. P values of ≤ 0.05, 0.01, 0.001 were considered as significant (*, **, ***).

## Results and discussion

### UPS-dependent degradation of nuclear RelA in *H. pylori* infection

**Fig. 1:**
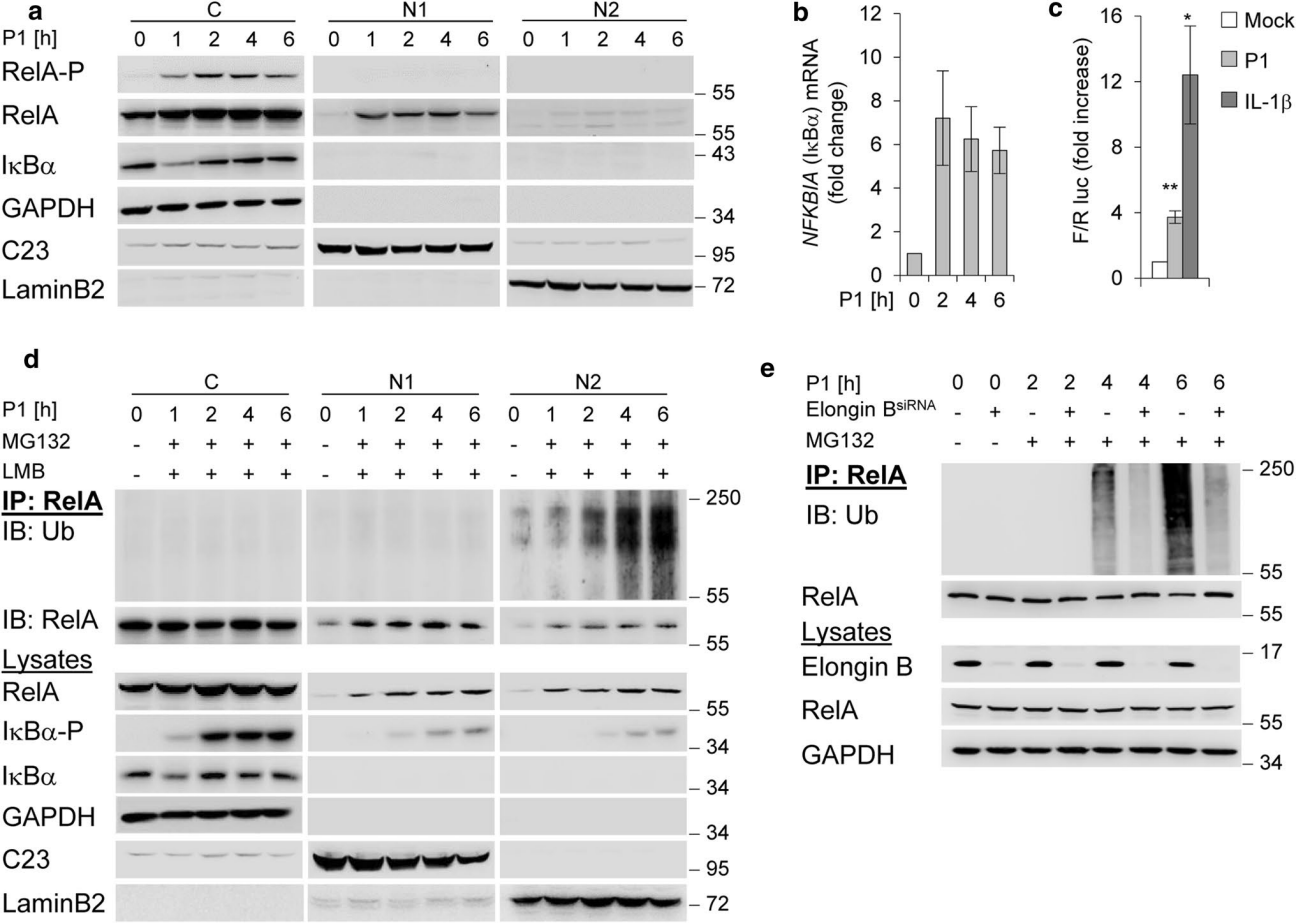
NF-κB/RelA turnover in *H. pylori* infection. **a** AGS cells were infected with *H. pylori* for the indicated times. Subcellular fractions were subjected to IB for analysis of the indicated proteins. **b** Total RNA was isolated from *H. pylori*-infected AGS cells and analysed using quantitative RT-PCR for the *NFKBIA* (the gene of IκB*α*) transcript. Data shown depict the average of triplicate determinations normalized to *GAPDH* housekeeping gene. Error bars denote mean ± SD. **c** AGS cells were transfected with luciferase reporters, treated with *H. pylori* or IL-1*β* (10 ng/ml) and fold increase of NF-κB transactivation activity (Firefly/Renilla luc) analysed after 3.5 h in a transactivation assay. **d** RelA-IP from subcellular fractions of *H. pylori*-infected AGS cells treated with MG132 and LMB 30 min after infection. The RelA-IP was performed at the indicated times in the presence of NEM and OPT, followed by IB analysis of the indicated proteins. **e** AGS cells were transfected with siRNA against Elongin B and infected with *H. pylori* for the indicated times (MG132 was added 30 min after infection). IP with an anti-RelA or isotype-matched antibody (IgG) was performed at the indicated times in the presence of NEM and OPT, followed by IB analysis of the indicated proteins. Data information: Data shown in (**a**, **d**, **e**) are representative for at least two independent experiments. Data shown in (**b**, **c**) are from one experiment with three technical replicates. GAPDH, C23 and LaminB2 served as load controls and indicate the purity of the subcellular fractions

The recently discovered *H. pylori* effector molecule ADP heptose induces classical NF-κB involving phosphorylation and subsequent proteasomal degradation of IκBα [4]. This leads to phosphorylation of RelA in the cytosol and its nuclear translocation within 1 h after *H. pylori* infection (Fig. 1a). Nuclear RelA induces mRNA expression of *NFKBIA* (Fig. 1b), and the de novo synthesised IκBα re-accumulated in the cytoplasm within 2 h (Fig. 1a). In addition, NF-κB transactivation activity was increased in cells infected with *H. pylori*, but to a lesser extent than in cells treated with IL-1β (Fig. 1c). For stabilisation of the de novo synthesised IκBα, it interacts with the CSN [26], which transmits the CSN-associated DUB USP15 [11] to the CRL-substrate IκBα to prevent it from degradation [14]. In addition, we investigated the effects of *H. pylori* infection on the turnover of nuclear RelA. We observed that the abundance of nuclear RelA in the soluble nuclear fraction (N1) and the fraction with subnuclear structures (N2) [10] decreases within 6 h of infection (Fig. 1a). Ubiquitinylation and proteasomal degradation of RelA regulate its stability and abundance and actively promote transcription termination [7]. We determined *H. pylori*-induced ubiquitinylation of RelA in subcellular fractions in the presence of MG132 and Leptomycin B (LMB) to prevent proteasomal degradation and nuclear export. Treatment of the cells with MG132 30 min after infection allowed phosphorylation and degradation of IκBα, and subsequently RelA translocation to the nucleus (Fig. 1d). Immunoprecipitation (IP) of RelA under denaturing conditions showed an enrichment of ubiquitinylated RelA (RelA-Ub) in the subnuclear fraction (N2) with increasing time (Fig. 1d). Ubiquitinylation of RelA is regulated by a multimeric ubiquitin-RING-ligase ECS^SOCS1^ containing cullin-2, the adaptor proteins elongin B and C, and substrate recognition molecule SOCS1 after TNF stimulation [9]. We therefore performed a transient knockdown of elongin B to investigate the effects of ECS^SOCS1^ on RelA ubiquitinylation induced by *H. pylori*. The result was that RelA ubiquitinylation induced by *H. pylori* infection was abolished in elongin B depleted cells (Fig. 1e), suggesting that ECS^SOCS1^ is the predominant E3 ligase for RelA degradation in *H. pylori* infection. So far, our data suggest that nuclear RelA induced by *H. pylori* is promptly regulated by ubiquitinylation and degradation through the proteasome.

### USP48 deubiquitinylates and counteracts UPS-dependent degradation of nuclear RelA in *H. pylori* infected cells

**Fig. 2:**
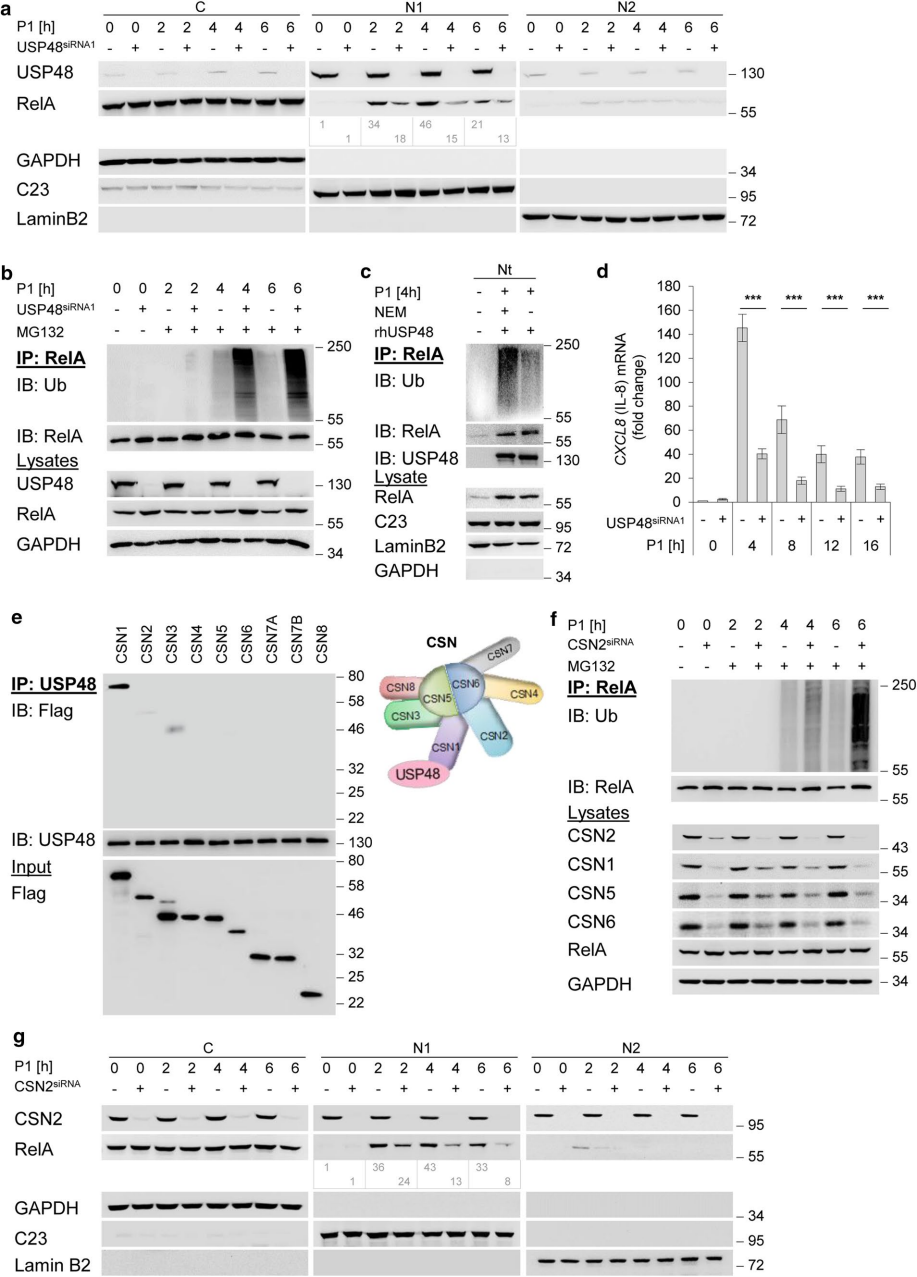
CSN-associated USP48 deubiquitinylates nuclear RelA. **a** AGS cells were transfected with siRNA against USP48 and infected with *H. pylori* for indicated times. Subcellular fractions were subjected to IB analysis of the indicated proteins. **b** AGS cells were transfected with siRNA against USP48 and infected with *H. pylori* for the indicated times (MG132 was added 30 min after infection). IP with an anti-RelA antibody or isotype-matched antibody (IgG) was performed at the indicated times in the presence of NEM and OPT, followed by IB analysis of the indicated proteins. **c** RelA-IP from the total nuclear fraction of *H. pylori*-infected AGS cells was incubated with recombinant human USP48 in the presence or absence of NEM, followed by IB analysis of the indicated proteins. **d** AGS cells were transfected with siRNA against USP48 and infected with *H. pylori* for the indicated times. Total RNA was isolated at the indicated times and analysed using quantitative RT-PCR for the *CXCL8* transcript (the gene of IL-8). Data shown depict the average of triplicate determinations normalized to *GAPDH* housekeeping gene. Error bars denote mean ± SD. **e** Equimolar amounts of in-vitro translated Flag-CSN subunits and 0.5 μg of recombinant human USP48 were incubated at 37 °C for 1 h. IP with an anti-USP48 antibody was performed, followed by IB analysis with anti-Flag and anti-USP48 antibodies. **f** AGS cells were transfected with siRNA against CSN2 and infected with *H. pylori* for the indicated times (MG132 was added 30 min after infection). IP with an anti-RelA antibody was performed at the indicated times in the presence of NEM and OPT, followed by IB analysis of the indicated proteins. **g** AGS cells were transfected with siRNA against CSN2 and infected with *H. pylori* for indicated times. Subcellular fractions were subjected to IB analysis of the indicated proteins. Data information: Data shown in (**a**–**c**, **e**–**g**) are representative for at least two independent experiments. Data shown in (d) are from three independent experiments with three technical replicates. ***P ≤ 0.001 (Student’s *t*-test). The band intensities shown in (**a**, **g**) were quantified using ImageJ software (NIH). GAPDH, C23 and LaminB2 served as load controls and indicate the purity of the subcellular fractions

We reported that the catalytic USP domain of USP48 interacts physically with the N-terminal region of the Rel homology domain (RHD) of RelA [27]. To analyse the impact of USP48 on nuclear RelA turnover in *H. pylori* infected cells, we performed a transient knockdown of USP48 in AGS and NCI-N87 cells. Our data show that the level of nuclear RelA decreased in USP48-depleted cells (Fig. 2a and Fig. S1), indicating a protective role of USP48 for nuclear RelA turnover in *H. pylori* infection. To detect ubiquitinylated nuclear RelA during *H. pylori* infection, we treated cells with the proteasome inhibitor MG132 30 min after infection. We observed enhanced accumulation of RelA-Ub within 4 h of *H. pylori* infection in USP48-depleted cells in a RelA-IP under denaturing conditions (Fig. 2b). In addition, we transiently transfected USP48 protein before *H. pylori* infection and observed removal of ubiquitin from RelA immunoprecipitated from cell lysates (Fig. S2). To determine whether USP48 directly deubiquitinylates nuclear RelA, we performed an in vitro DUB assay in which we incubated ubiquitinylated RelA immunoprecipitated from the nuclear fraction of cells infected with *H. pylori* together with recombinant USP48. We found that USP48 cleaved poly-Ub-chains bound to RelA. The presence of the DUB inhibitor N-ethylmaleimide (NEM) abolished the deubiquitinylation of RelA (Fig. 2c), suggesting that USP48 deubiquitinylates nuclear RelA. Furthermore, we observed lower expression of the NF-κB target gene *CXCL8* in *H. pylori* infection in USP48-depleted cells (Fig. 2d), consistent with increased degradation and lower nuclear accumulation of RelA (Fig. 2a). Accordingly, our data suggest that USP48 stabilises and prolongs the transcriptional activity of NF-κB/RelA. Several reports have suggested a role of USP48 in protein stability in diverse cellular processes, e.g. glioma-associated oncogene 1 (Gli1) in glioblastoma [28], TRAF2 in epithelial barrier dysfunction [29] and antagonising Breast Cancer 1 (BRCA1) E3 ligase on histone H2A and genome stability [30]. Notably, USP48 could also promote the stability of oncoprotein mouse double minute 2 (Mdm2) in a deubiquitinylation-independent manner [31]. Recently, USP48 was reported to stabilise SIRT6 by K48-linked deubiquitinylation, which impedes metabolic reprogramming in hepatocellular carcinoma (HCC) [32], and control cell cycle progression by stabilising the Aurora B protein [33].

### Stabilisation of nuclear RelA in *H. pylori* infection is CSN-dependent

USP48 has been reported to interact with the CSN [15], which function as a signalling platform and integrates the activity of several proteins [11]. To identify the direct physical interaction partner within the CSN complex, we translated each CSN subunit in vitro and performed an IP with recombinant USP48. In this way, we identified CSN1 as the CSN subunit that interacts with USP48 (Fig. 2e). Interestingly, we previously observed that CSN1 also interacts with IκB*α* [26]. The only other subunit interacting with IκB*α* is CSN3, which showed also a weak interaction with USP48 suggesting that the juxtaposed CSN1 and CSN3 cooperate in recruiting USP48 [26]. Further, it was reported that CSN1 interacts with a number of other molecules, such as deneddylase 1 (DEN1), a cysteine protease in the Ub-like protease (ULP) family possessing deneddylase activity. CSN1-DEN1 interaction initiates DEN1 degradation, thereby balancing cellular deneddylase activity [34]. In addition, it was reported that CSN1 interacts with inositol 1,3,4-trisphosphate 5/6-kinase [35], Ankyrin repeat and SOCS box containing protein 4 [6], SAP130/SF3b-3 [36], and Tonsoku-associating protein 1 [37]. To determine the role of the CSN in nuclear RelA turnover, we performed a transient knockdown of the CSN2 subunit, which impairs the integrity of the CSN [38]. Downregulation of single CSN subunits by siRNA caused a significant and coordinated reduction of other CSN subunits suggesting a coordinated expression of the subunits tightly linked to the CSN complex assembly. As a common post-transcriptional regulator for CSN subunits, miRNAs of the let-7 family have been identified because a reduction or block of let-7 miRNAs induces the coordinated expression of CSN subunits [39]. Corresponding to USP48-depleted cells, we observed an increased accumulation of RelA-Ub (Fig. 2f) and a decrease of nuclear RelA in the CSN2 depleted cells (Fig. 2g).

### USP48 controls A20 de novo synthesis and suppresses caspase cleavages

**Fig. 3:**
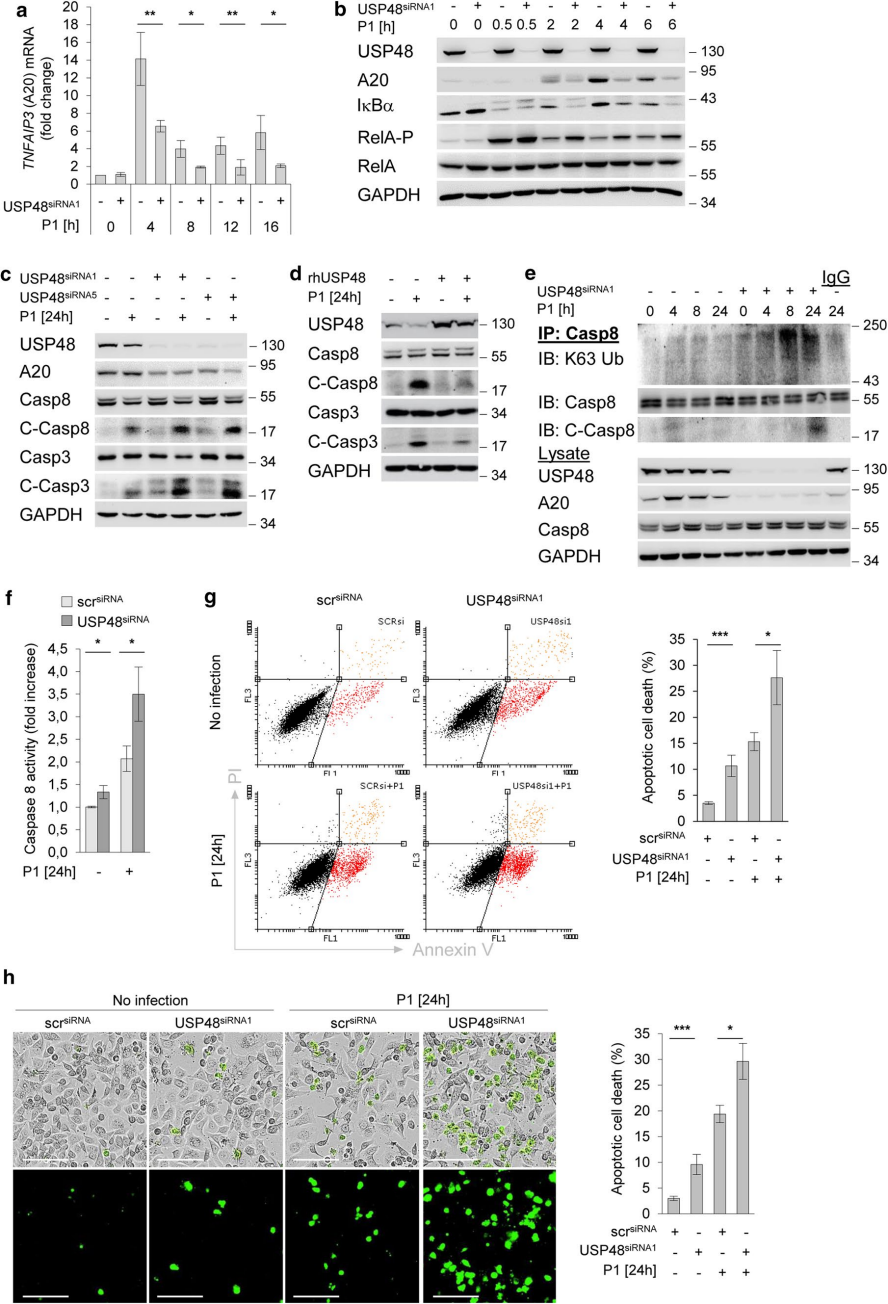
USP48 controls A20 de novo synthesis and suppresses caspase cleavages. **a** AGS cells were transfected with siRNA against USP48 and infected with *H. pylori* for the indicated times. Total RNA was isolated at the indicated times and analysed using quantitative RT-PCR for the *TNFAIP3* transcript (gene of A20). Data shown depict the average of triplicate determinations normalized to *GAPDH* housekeeping gene. Error bars denote mean ± SD. **b**, **c** AGS cells were transfected with siRNA against USP48 and infected with *H. pylori* for indicated times. Whole-cell lysates were subjected to IB for analysis of the indicated proteins. C-Casp8 or C-Casp3 = cleaved caspases. **d** AGS cells were transfected with recombinant human USP48 and infected with *H. pylori* for 24 h. Whole-cell lysates were subjected to IB analysis of the indicated proteins. **e** AGS cells were transfected with siRNA against USP48 and infected with *H. pylori* for the indicated times. IP with an anti-Caspase-8 antibody was performed at the indicated times in the presence of NEM and OPT, followed by IB analysis of the indicated proteins. **f** AGS cells transfected with siRNA were infected with *H. pylori* for 24 h before incubation with Caspase-Glo^®^ 8 reagent for 1 h. Luminescence of caspase-8 activity was measured and calculated as a fold increase compared to the uninfected scramble control. **g** AGS cells were transfected with siRNA against USP48 and infected with *H. pylori* for 24 h, followed by staining with annexin V/PI. Apoptotic cell death was analysed by flow cytometry. Data shown depict the average of two independent experiments. Error bars denote mean ± SD. **h** AGS cells were transfected with siRNA against USP48 and infected with *H. pylori* for 24 h. Cleaved caspase-3/7 expression was detected by the IncuCyte^®^ S3 Live-Cell Analysis Image system. Scale bars = 100 µm. Data shown depict the average of four pictures from distinct regions. Error bars denote mean ± SD. Data information: Data shown in (**a**) are from three independent experiments with three technical replicates. Data shown in (**b**–**e**, **g**) are representative for at least two independent experiments. Data shown in (**f**) are from one experiment with three technical replicates. Data shown in (**h**) are from one experiments with four technical replicates. *P ≤ 0.05, **P ≤ 0.01, ***P ≤ 0.001 (Student’s *t*-test)

We previously reported NF-κB-dependent upregulation of A20 in *H. pylori* infection [21]. As expected we found in *H. pylori* infection that induction of NF-κB-dependent A20 de novo synthesis was suppressed in USP48-depleted cells, in both mRNA (Fig. 3a) and protein levels (Fig. 3b and Fig. S3a). In addition, we show that *H. pylori* strain (P12) similarly regulates USP48-dependent control of A20 in AGS cells (Fig. S3b). Notably, we reported that A20 inhibits apoptotic cell death by deubiquitinylating caspase-8, interfering with the caspase-8 activation [21]. The K63-linked polyubiquitinylation of caspase-8 mediates its processing into a fully activated cleaved form that subsequently mediates cleavage of the critical exogenous apoptotic substrates, including caspase-3 and -7 [40]. To investigate the relationship between caspase cleavage and apoptotic cell death in *H. pylori*-infected cells, we analysed cells in which caspase-8 was knocked down and showed suppression of caspase-3 cleavage (Fig. S4a) and apoptotic cell death (Fig. S4b). Similar results were observed when *H. pylori*-infected cells were treated with the caspase-8 specific inhibitor Z-IETD-FMK [21]. We investigated the effects of USP48 on the activation of apoptotic caspases in *H. pylori* infection. An increase in activated cleaved caspase-8 and -3 was observed in USP48-depleted cells (Fig. 3c). Conversely, transient transfection of USP48 protein before *H. pylori* infection suppressed cleavage of caspases (Fig. 3d). Therefore, we hypothesised that USP48 counteracts the ubiquitinylation of caspase-8 in an A20-dependent manner. IP of caspase-8 under denaturing conditions showed an increase in K63-linked polyubiquitinylation of caspase-8 in USP48-depleted cells, corresponding to an accumulation of cleaved caspase-8 (Fig. 3e). Moreover, we performed Caspase-Glo8 Assay and observed that caspase-8 activity was increased in USP48 knockdown cells (Fig. 3f). These data suggest that USP48 can promote deubiquitinylation of caspase-8 and impair caspase-8 activity. We also investigated the effects of USP48 on *H. pylori* induced apoptotic cell death in gastric epithelial cells. Analysis of annexin V/PI stained cells showed increased apoptotic cell death after deletion of USP48 (Fig. 3g and Fig. S5). Accordingly, *H. pylori* infection induces apoptotic cell death in various cell lines via a pathway that involves sequential induction of caspase activity. In addition, we studied *H. pylori* associated apoptotic cell death by detection of caspase-3/7-stained cells using Incucyte^®^ Live-Cell Imaging analysis and showed more apoptotic cell death in USP48-depleted cells (Fig. 3h).

### USP48-suppressed apoptotic cell death is A20-dependent

**Fig. 4:**
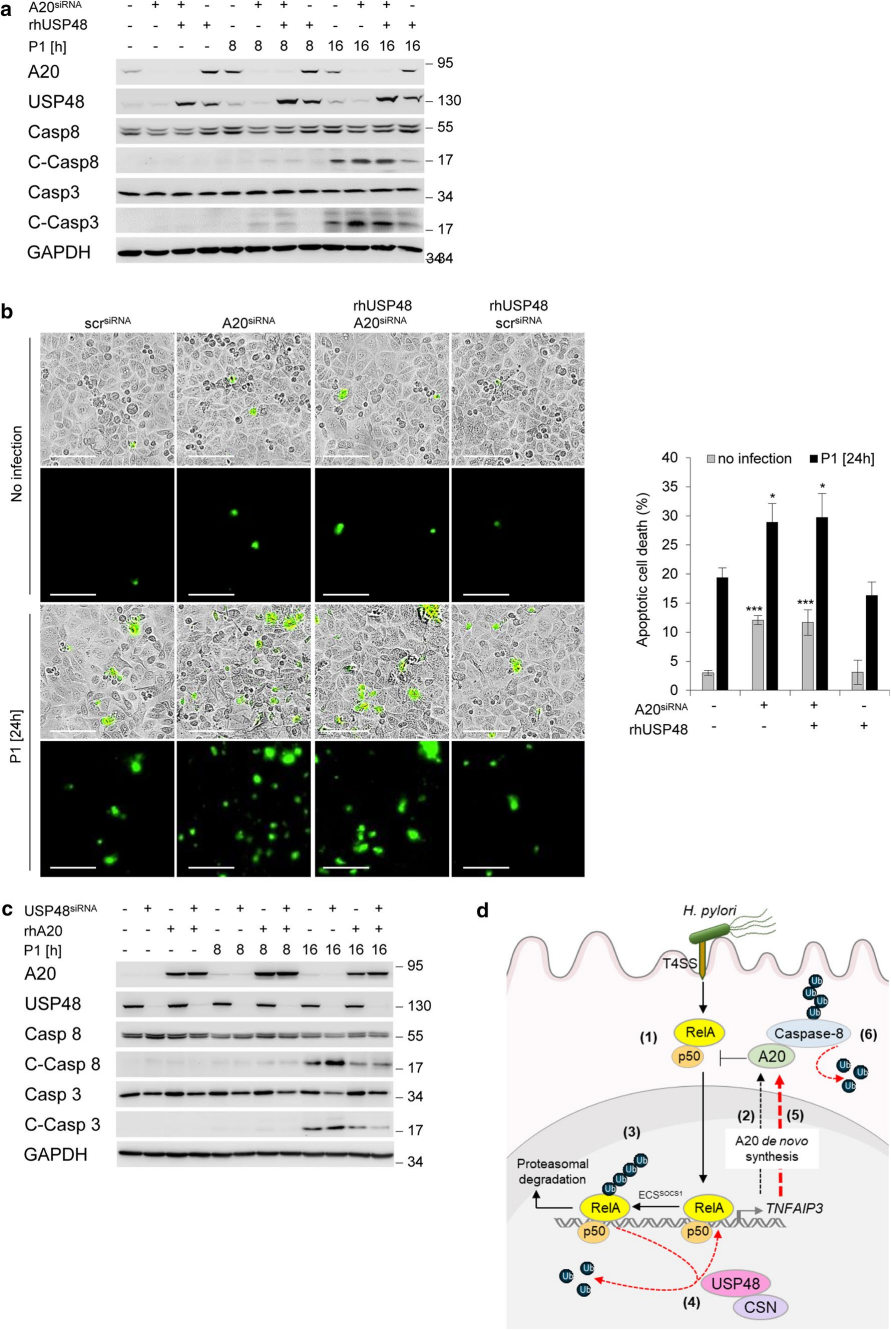
USP48-suppressed caspase cleavage is A20-dependent. **a** AGS cells were transfected with siRNA against A20, and subsequently transfected with recombinant human USP48. One hour after transfection, the cells were infected with *H. pylori* for indicated times. Whole-cell lysates were subjected to IB for analysis of the indicated proteins. **b** AGS cells were transfected with siRNA against A20, and subsequently transfected with recombinant human USP48. One hour after transfection, the cells were infected with *H. pylori* for 24 h. Cleaved caspase-3/7 expression was detected by the IncuCyte^®^ S3 Live-Cell Analysis Image system. Scale bars = 100 µm. Data shown depict the average of four pictures from distinct regions. Error bars denote mean ± SD. **c** AGS cells were transfected with siRNA against USP48 and then transfected with recombinant human A20 protein. One hour after protein transfection, cells were infected with *H. pylori* for the indicated times. Total cell lysates were subjected to IB analysis of the indicated proteins. **d** Schematic representation of the findings in this study. Infection of *H. pylori* induces fast activation of NF-κB, leading to nuclear translocation of RelA (1) and expression of the target gene *TNFAIP3* (encodes for A20) (2). Termination of RelA activity by ECS^socs1^-dependent ubiquitinylation and degradation (3). CSN-associated USP48 deubiquitinylates RelA-Ub resulting in RelA stabilisation (4) and prolonged A20 de novo synthesis (5). USP48 and A20 synergistically suppresses caspase-8 activity and apoptotic cell death (6). Data information: Data shown in (**a**, **c**) are representative for at least two independent experiments. Data shown in (**b**) are from one experiments with four technical replicates. *P ≤ 0.05, ***P ≤ 0.001 (Student’s *t*-test)

The effects of USP48 on A20 expression and caspase-8 cleavage and apoptotic cell death in cells infected with *H. pylori* strongly suggest that the effect is dependent on A20. To test this hypothesis, we transiently transfected recombinant USP48 protein into A20 knockdown cells and found that USP48 rescued *H. pylori*-induced caspase cleavages in parental cells but not in A20-depleted cells (Fig. 4a). A similar result was obtained when we analysed apoptotic cells stained for caspase-3/7 by Incucyte^®^ Live-Cell Imaging (Fig. 4b). Furthermore, transfection of A20 protein in USP48-depleted cells before *H. pylori* infection suppressed caspase cleavage and apoptotic cell death (Fig. 4c), indicating that the protective effect on apoptotic cell death is dependent on the expression of A20. On the other hand, *H. pylori* infection induces reactive oxygen species (ROS) leading to apoptotic cell death, independent of the T4SS [17]. Gamma-glutamyl transpeptidase (GGT) is an effector that contributes to the production of H_2_O_2_ by glutathione (GSH) hydrolysis [41]. Future studies focussed on elucidating the *H. pylori*-associated pathogenesis of gastric disease will uncover the importance of apoptotic cell death for the mucosal microenvironment to better understand how this might contribute to gastric diseases.

Our study has shown that CSN-associated USP48 stabilises nuclear RelA through deubiquitinylation, thereby promoting the transcriptional activity of RelA to prolong A20 de novo synthesis. Furthermore, USP48 promotes cell survival during *H. pylori* infection through A20-dependent suppression of caspases activity and apoptotic cell death. We highlight the interplay of USP48 and A20 in regulating NF-κB and cell death in the gastric epithelium. This may contribute to the control of immune responses and gastric cancer development and represent an attractive target for future therapeutic intervention strategies.

### Supplementary Information

Below is the link to the electronic supplementary material.

Supplementary file1 (PDF 4288 KB)

## Data Availability

No data were deposited in a public database. Inquiries for reagents used in this study should be addressed to M. Naumann (naumann@med.ovgu.de).
