# Supplementary material for: USP48 and A20 synergistically promote cell survival in Helicobacter pylori infection
Source: Cell Mol Life Sci. 2022 Aug 1;79(8):461. doi: 10.1007/s00018-022-04489-7 (PMC9343311; doi:10.1007/s00018-022-04489-7)

Supplementary material

USP48 and A20 synergistically promote cell survival in *Helicobacter pylori*-infection

Phatcharida Jantaree[1], Supattra Chaithongyot[1], Olga Sokolova[1], Michael Naumann[1*]

[1]Otto von Guericke University, Institute of Experimental Internal Medicine, Medical Faculty, Leipziger Str. 44, 39120 Magdeburg, Germany

Figure legends

Figure S1. USP48 stabilises nuclear RelA in *H. pylori* infection
a NCI-N87 cells were transfected with siRNA[1], or **b** siRNA[5] against USP48 and infected with *H. pylori* for indicated times. Subcellular fractions were subjected to IB for analysis of the indicated proteins. Data information: Data shown are representative for at least two independent experiments. The band intensity was quantified using ImageJ software (NIH). GAPDH, C23 served as load controls and indicate for the purity of the subcellular fractions.

Figure S2. USP48 deubiquitinylates RelA
AGS cells were transfected with recombinant human USP48 (rhUSP48) and infected with *H. pylori* for the indicated times. MG132 was added 30 min after infection. IP with an anti-RelA antibody was performed at the indicated times in the presence of NEM and OPT, followed by IB analysis of the indicated proteins. Data information: Data shown are representative of at least two independent experiments.

Figure S3. USP48 prolongs A20 *de novo* synthesis
a NCI-N87 cells were transfected with siRNA against USP48 and infected with *H. pylori* for indicated times. **b** AGS cells were transfected with siRNA against USP48 and infected with *H. pylori* strain P12 for indicated times. Whole-cell lysates were subjected to IB for analysis of the indicated proteins. Data information: Data shown are representative for at least two independent experiments.

Figure S4. Caspase-8 cleavage contributes to H. *pylori* associated apoptotic cell death
a AGS cells were transfected with siRNA against caspase-8 and infected with *H. pylori* for indicated times. Whole-cell lysates were subjected to IB for analysis of the indicated proteins. **b** AGS cells were transfected with siRNA against caspase-8 and infected with *H. pylori* for 24 h. Cleaved caspase-3/7 expression was detected by the IncuCyte® S3 Live-Cell Analysis System. Scale bars = 100 µm. Data shown depict the average of nine pictures from distinct regions. Error bars denote mean ± SD. Data information: Data shown in (a) are representative for at least two independent experiments. Data shown in (b) are from two independent experiments. $^*P \leq 0.05$ (Student's t-test).

Figure S5. USP48 suppresses apoptotic cell death
AGS cells were transfected with siRNA against USP48 (siRNA[5]) and infected with *H. pylori* for 24 h, followed by staining with annexin V/PI. Apoptotic cell death was analysed by flow cytometry. Data shown depict the average of two independent experiments. Error bars denote mean ± SD. Data information: Data shown are representative for at least two independent experiments. $^*P \leq 0.05$, $^{**}P \leq 0.01$ (Student's t-test).

Figure S1

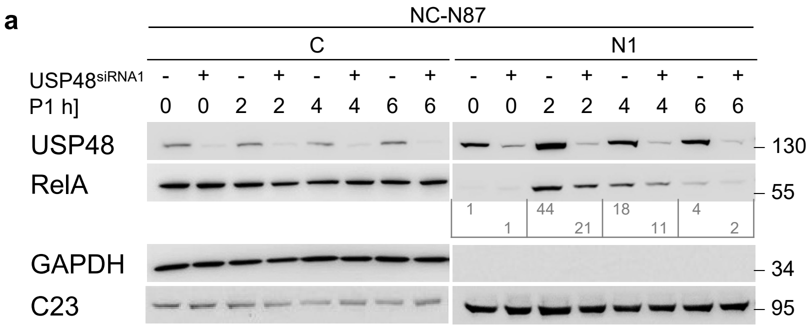
a
NC-N87
C
N1
USP48siRNA1
P1 h]
USP48
RelA
GAPDH
C23
130
55
34
95
1
1
44
21
18
11
4
2

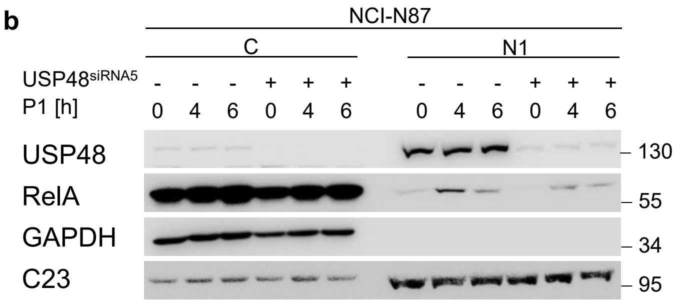
b
NCI-N87
C
N1
USP48siRNA5
P1 [h]
USP48
RelA
GAPDH
C23
130
55
34
95

Figure S2

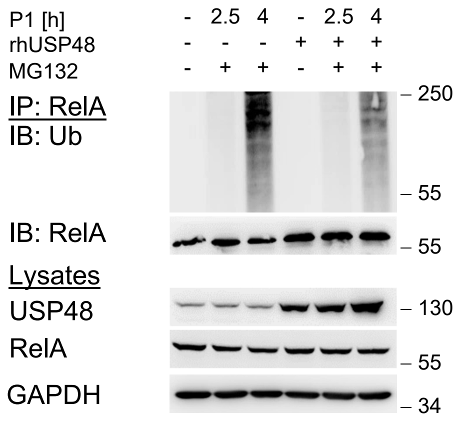
P1 [h]
- 2.5 4 - 2.5 4
rhUSP48
- - - + + +
MG132
- + + - + +
IP: RelA
IB: Ub
250
55
IB: RelA
55
Lysates
USP48
130
RelA
55
GAPDH
34

Figure S3

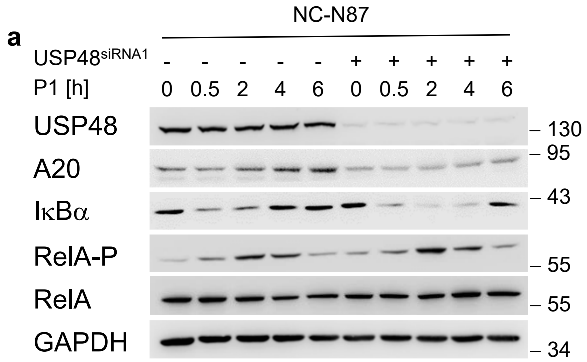
a
NC-N87
USP48siRNA1 - - - - - + + + + +
P1 [h] 0 0.5 2 4 6 0 0.5 2 4 6
USP48
A20
IκBα
RelA-P
RelA
GAPDH
130
95
43
55
55
34
b
USP48siRNA1 - + - + - + - + - +
P12 [h] 0 0 1 1 2 2 4 4 6 6
USP48
A20
IκBα
RelA-P
RelA
GAPDH
130
95
43
55
55
34

Figure S4

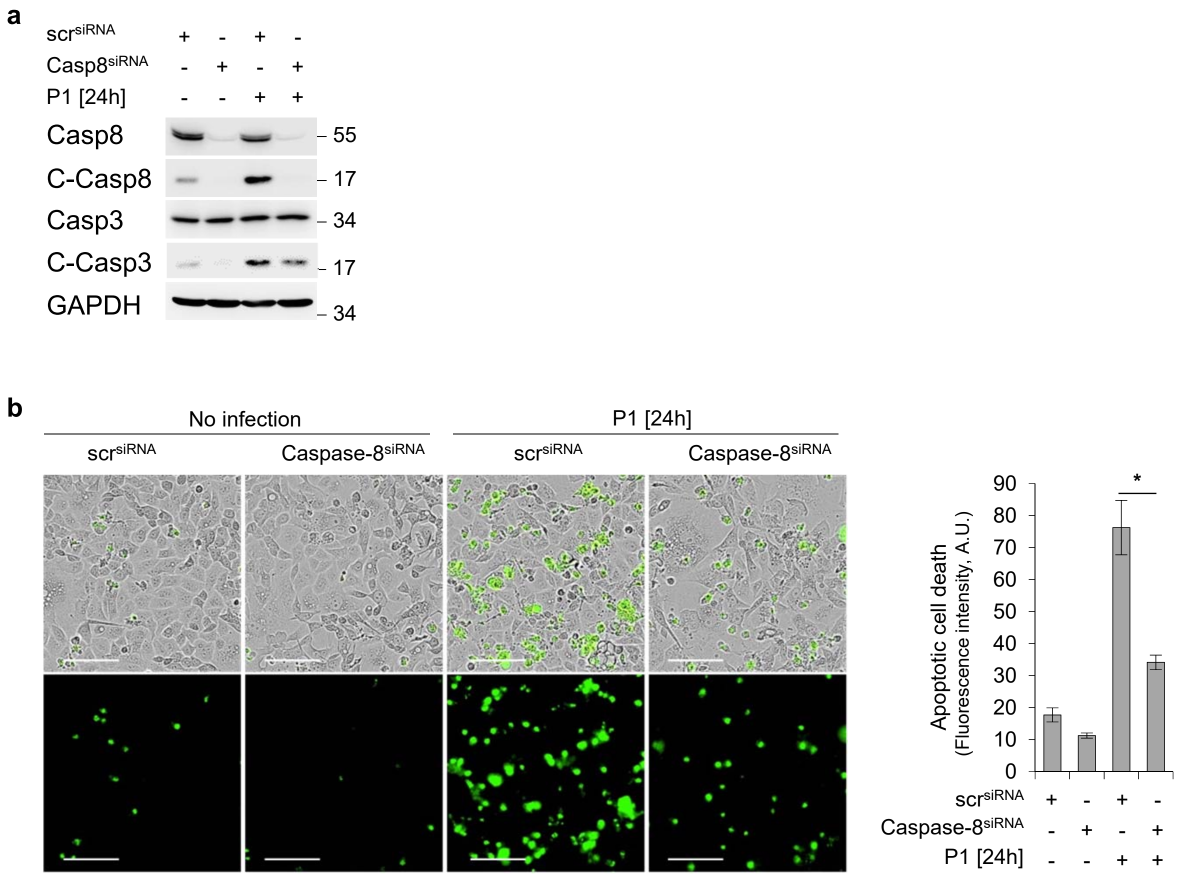
a
scr^siRNA + - + -
Casp8^siRNA - + - +
P1 [24h] - - + +
Casp8
C-Casp8
Casp3
C-Casp3
GAPDH
55
17
34
17
34
b
No infection
P1 [24h]
scr^siRNA
Caspase-8^siRNA
scr^siRNA
Caspase-8^siRNA
Apoptotic cell death
(Fluorescence intensity, A.U.)
90
80
70
60
50
40
30
20
10
0
*
scr^siRNA + - + -
Caspase-8^siRNA - + - +
P1 [24h] - - + +

Figure S5

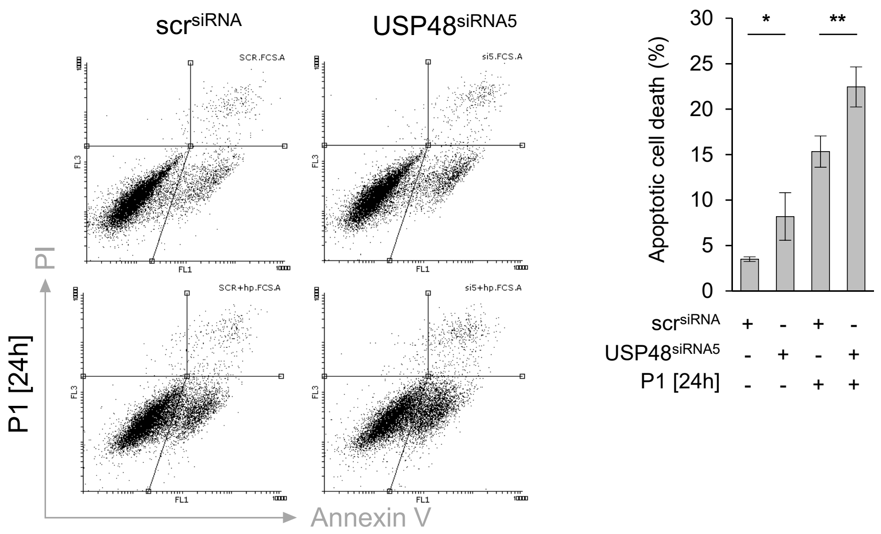
scr siRNA
USP48 siRNA5
SCR.FCS.A
si5.FCS.A
SCR+hp.FCS.A
si5+hp.FCS.A
FL3
FL1
PI
P1 [24h]
Annexin V
Apoptotic cell death (%)
30
25
20
15
10
5
0
*
**
scr siRNA + - + -
USP48 siRNA5 - + - +
P1 [24h] - - + +

Uncropped blot for Fig 1a

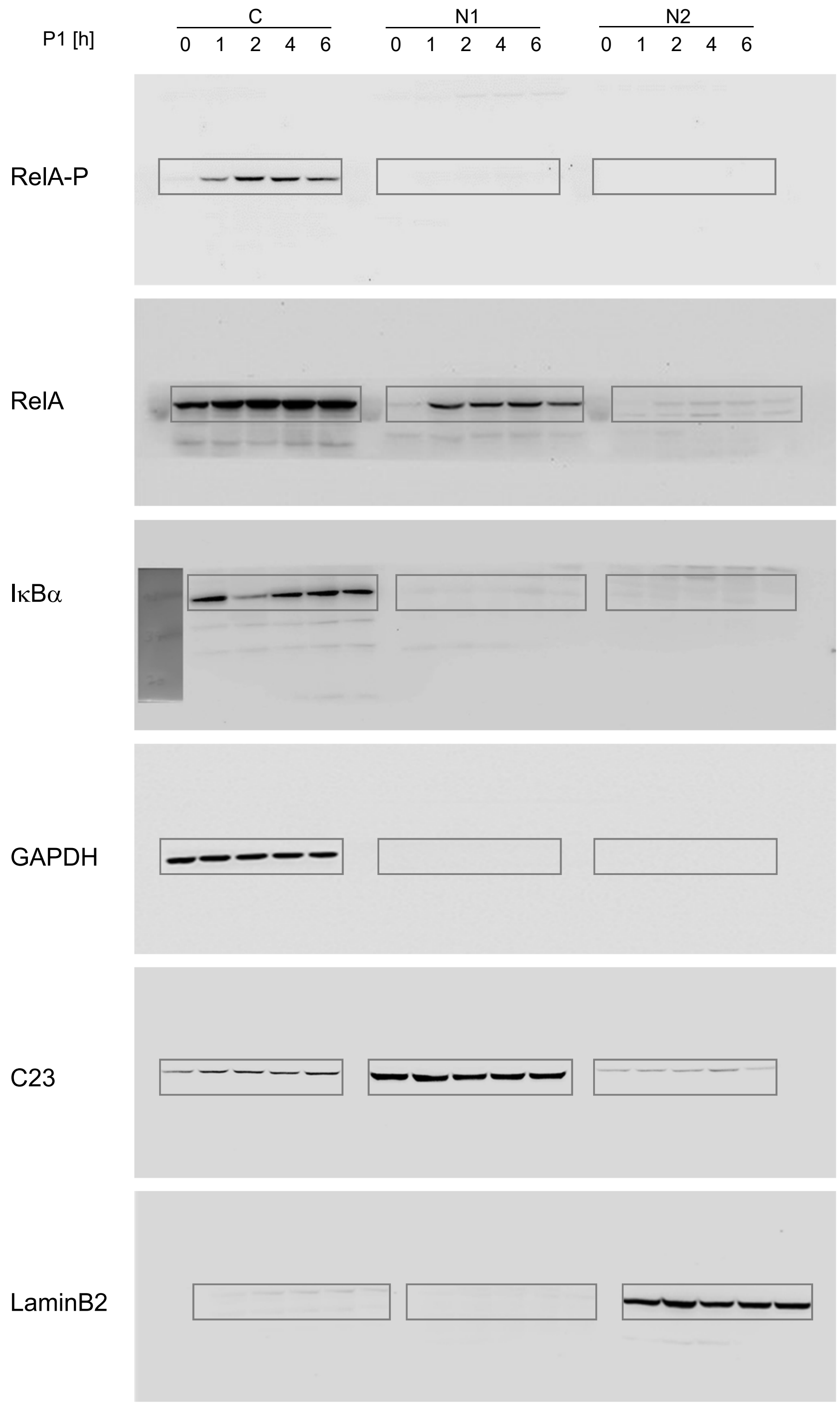

Uncropped blot for Fig 1d

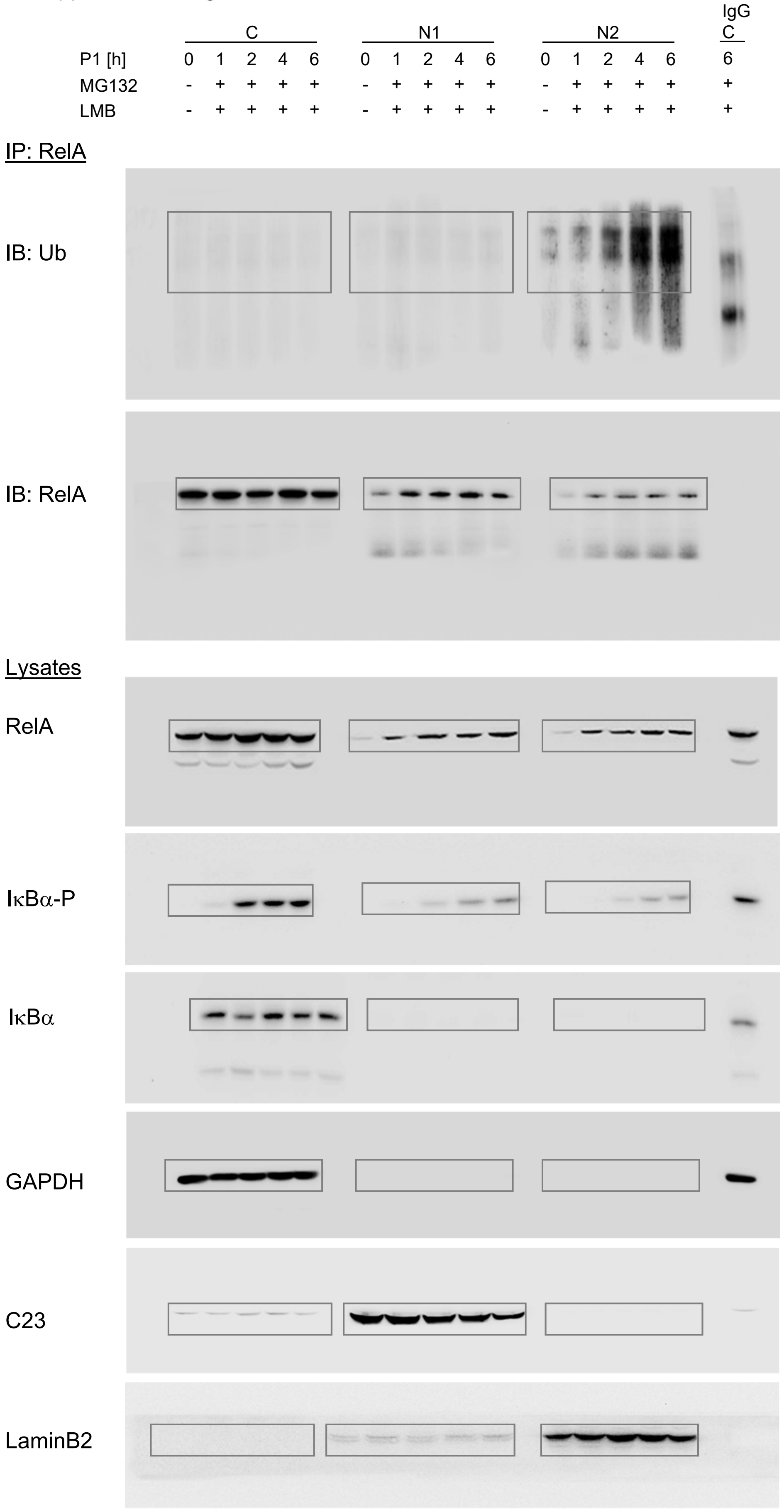

Uncropped blot for Fig 1e

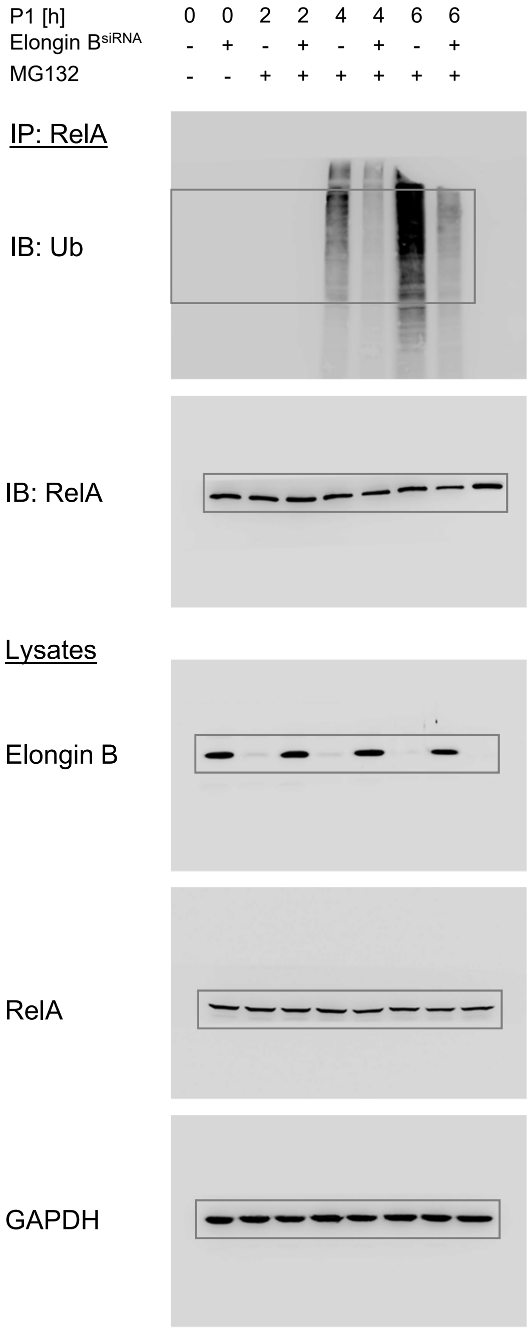

Uncropped blot for Fig 2a

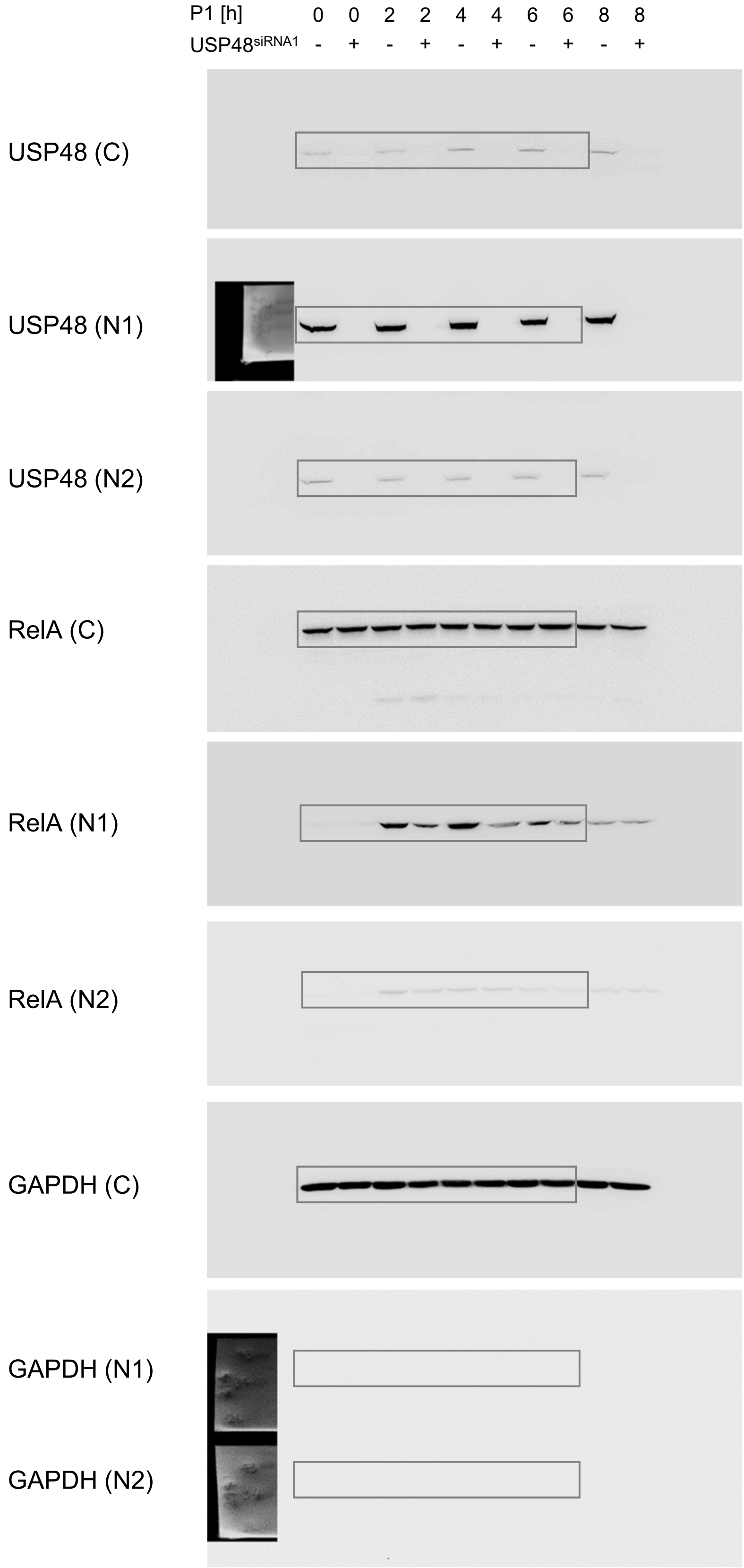

Uncropped blot for Fig 2a

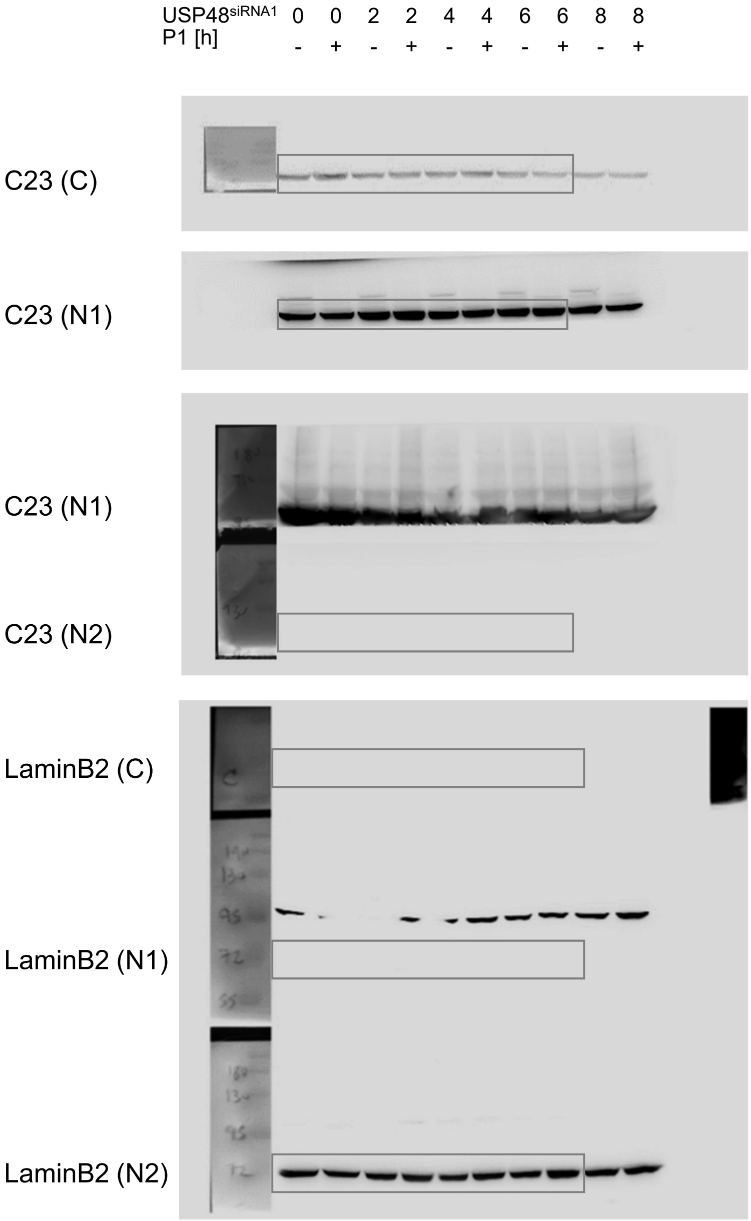

Uncropped blot for Fig 2b

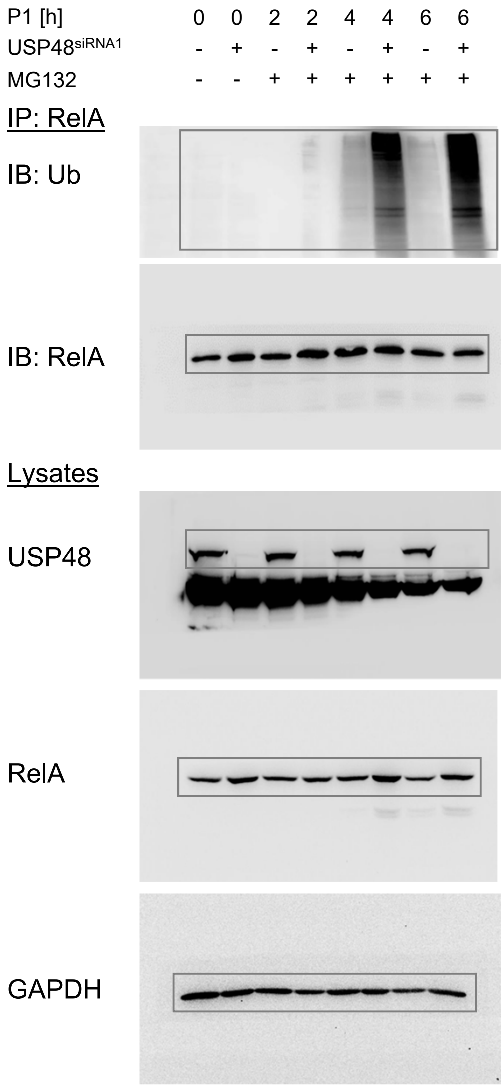

Uncropped blot for Fig 2c

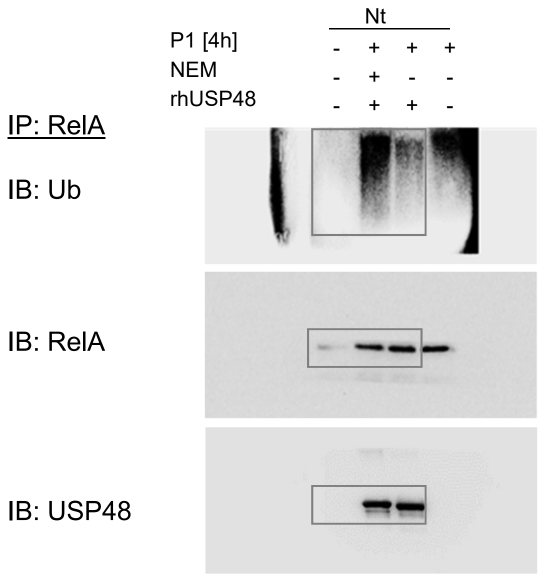

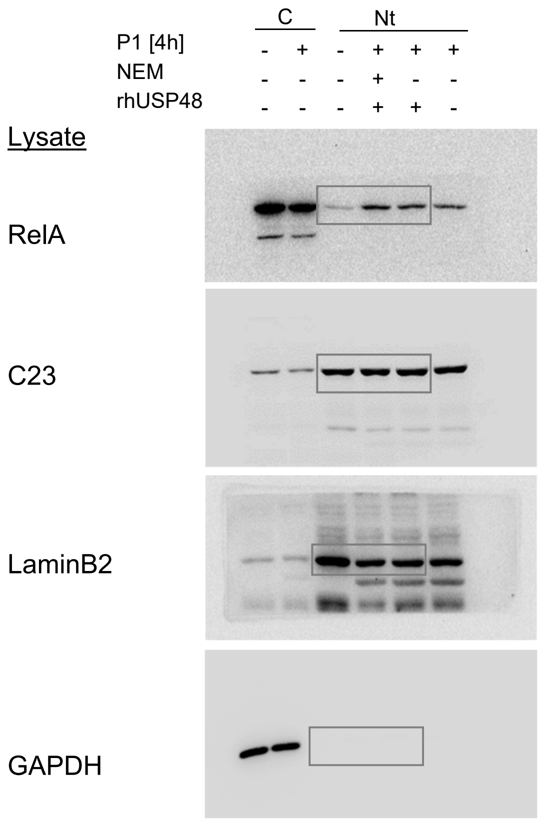

Uncropped blot for Fig 2e

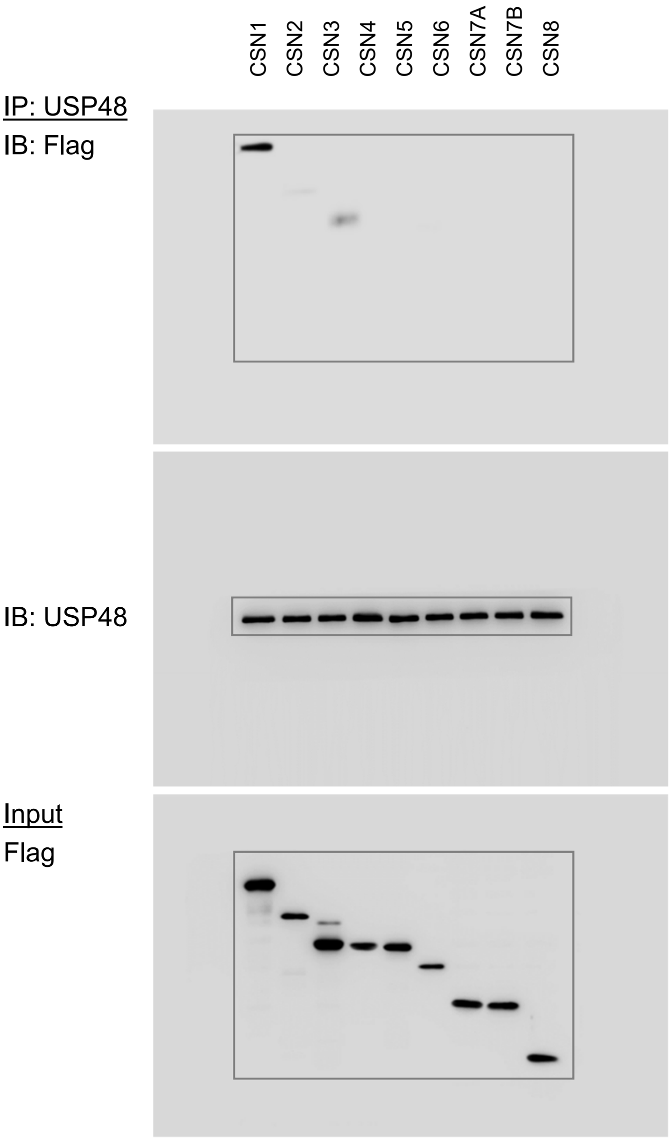

Uncropped blot for Fig 2f

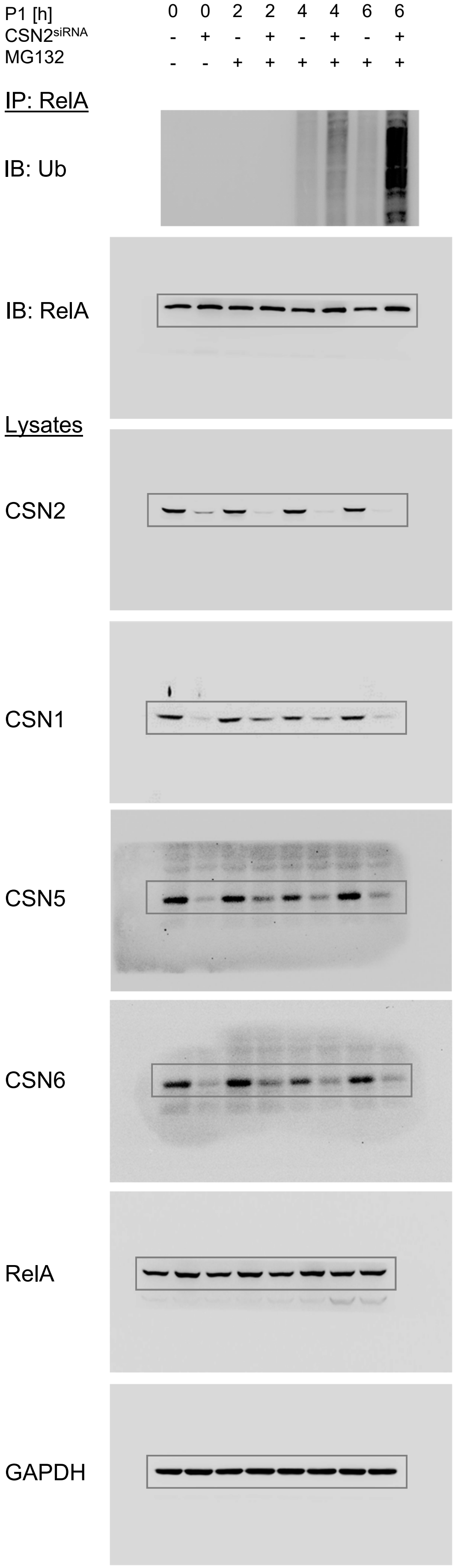

Uncropped blot for Fig 2g

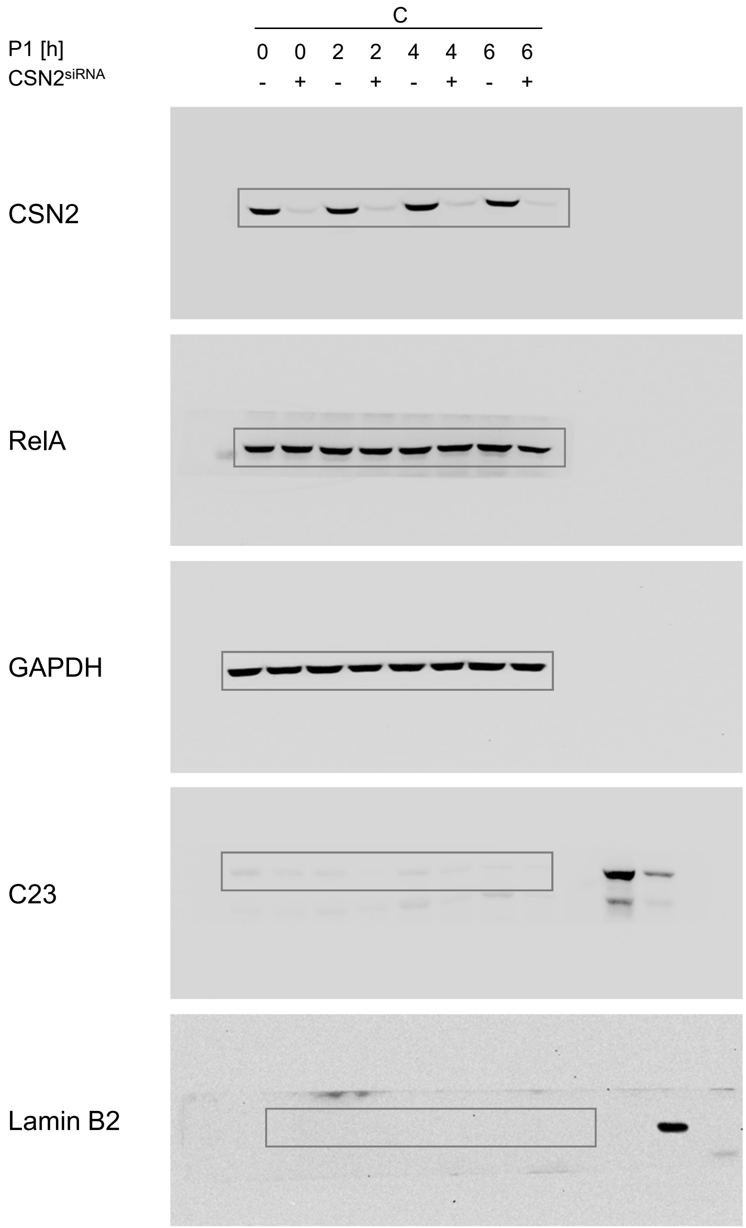

Uncropped blot for Fig 2g

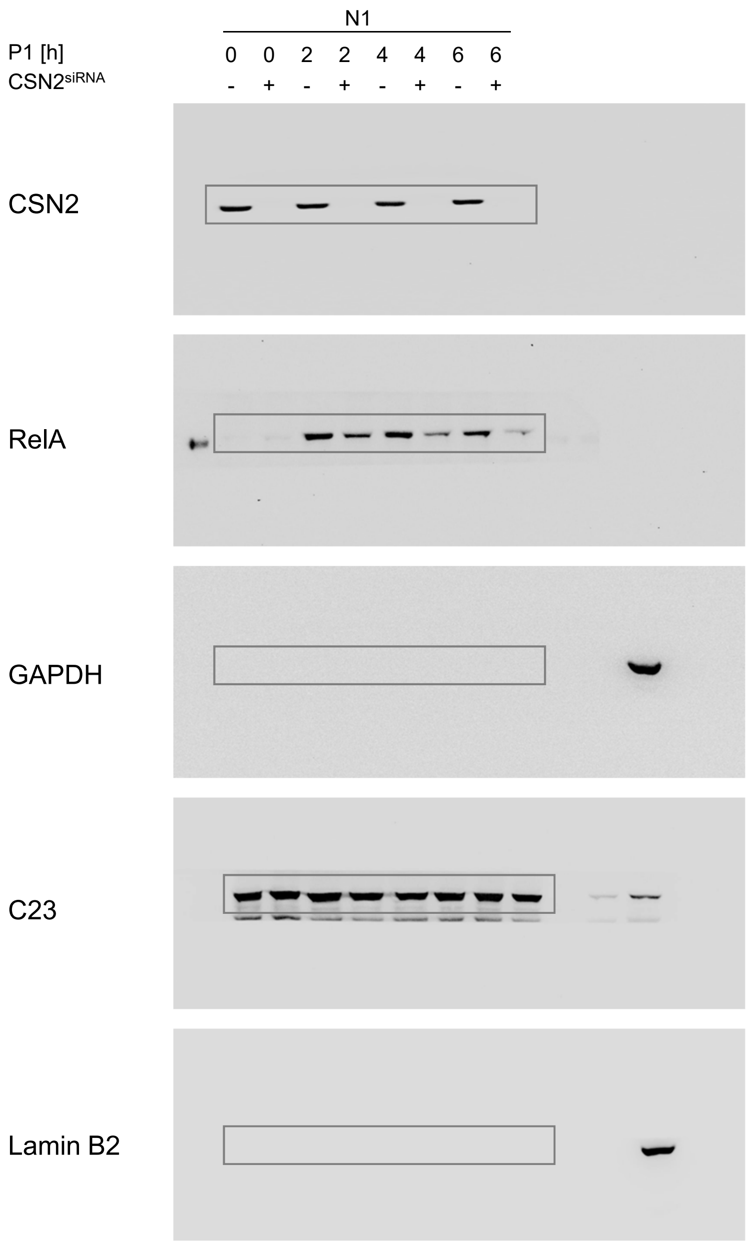

Uncropped blot for Fig 2g

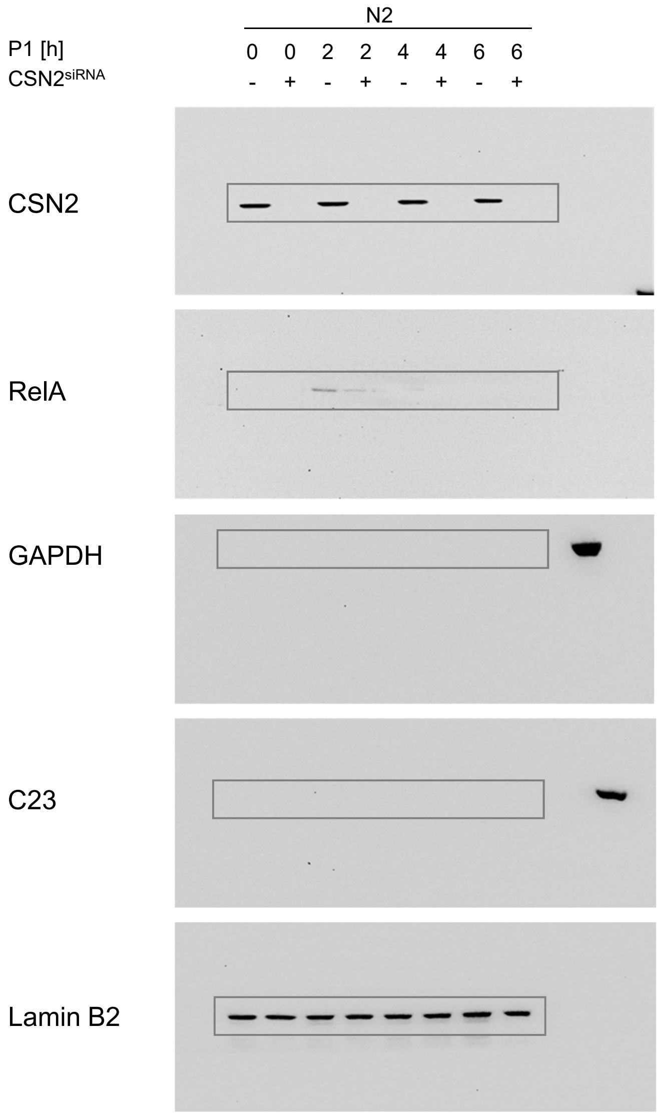

Uncropped blot for Fig 3b

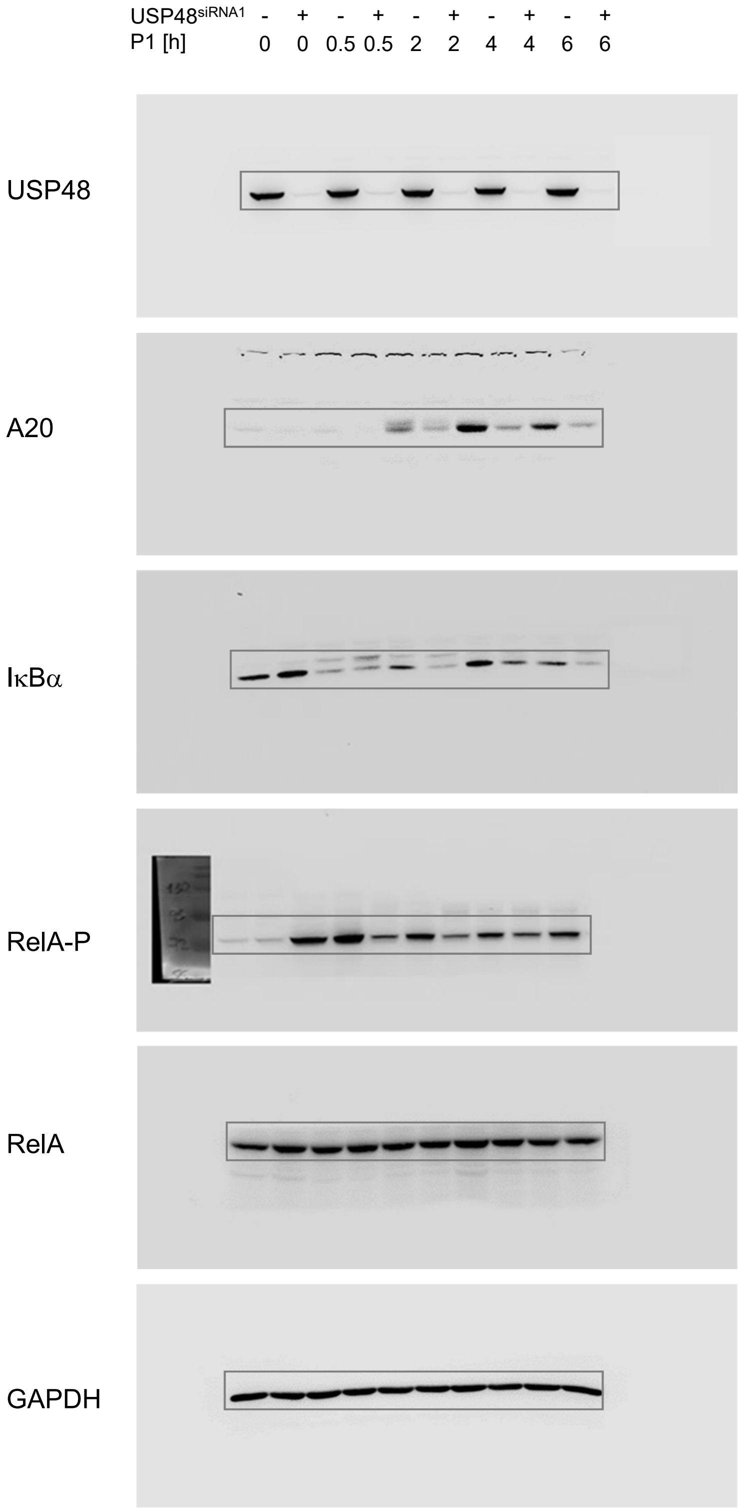

Uncropped blot for Fig 3c

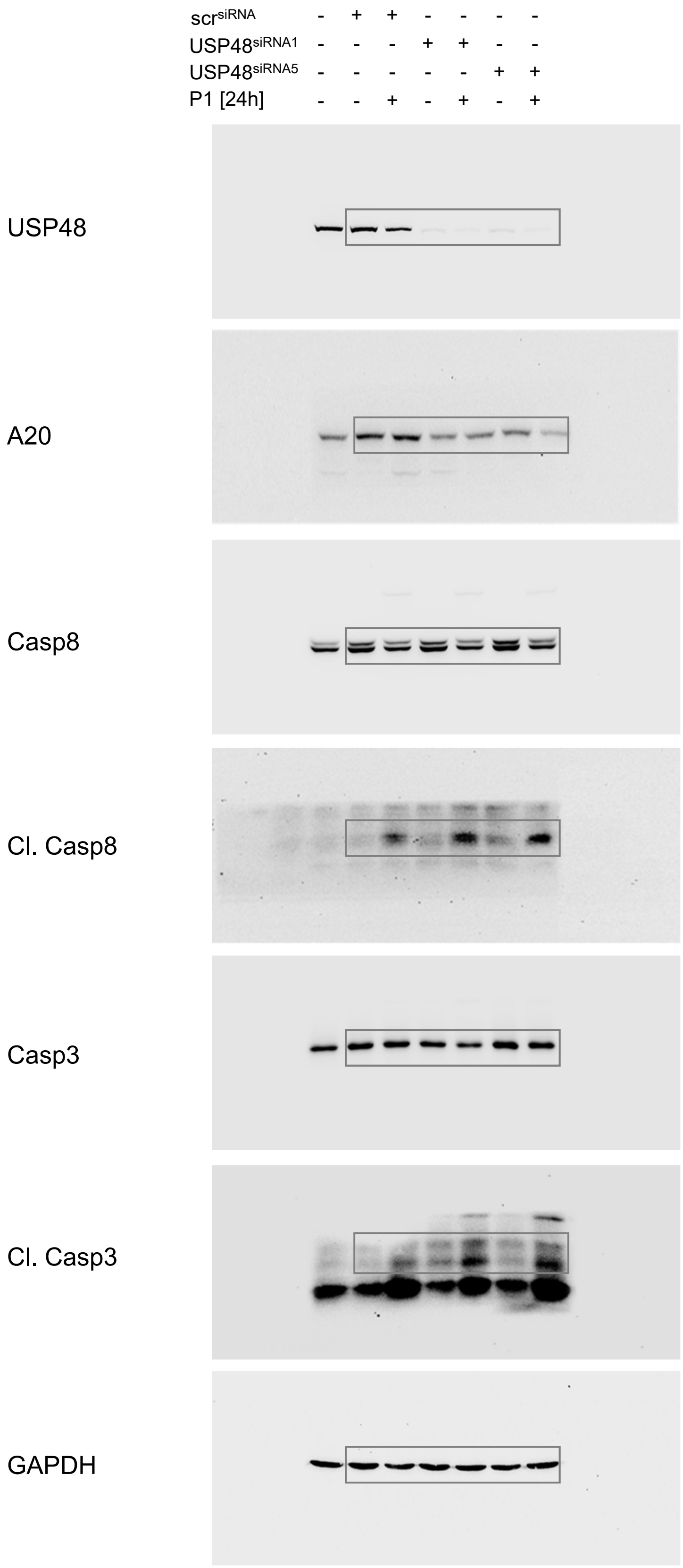

Uncropped blot for Fig 3d

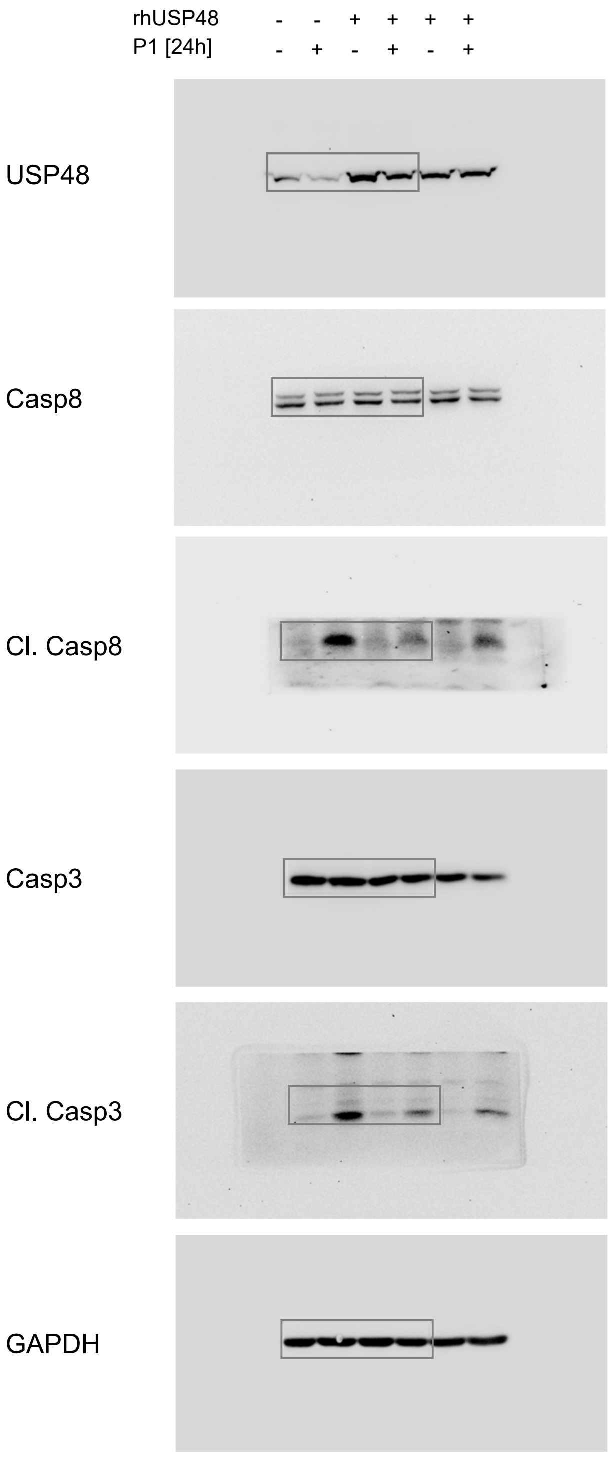

Uncropped blot for Fig 3e

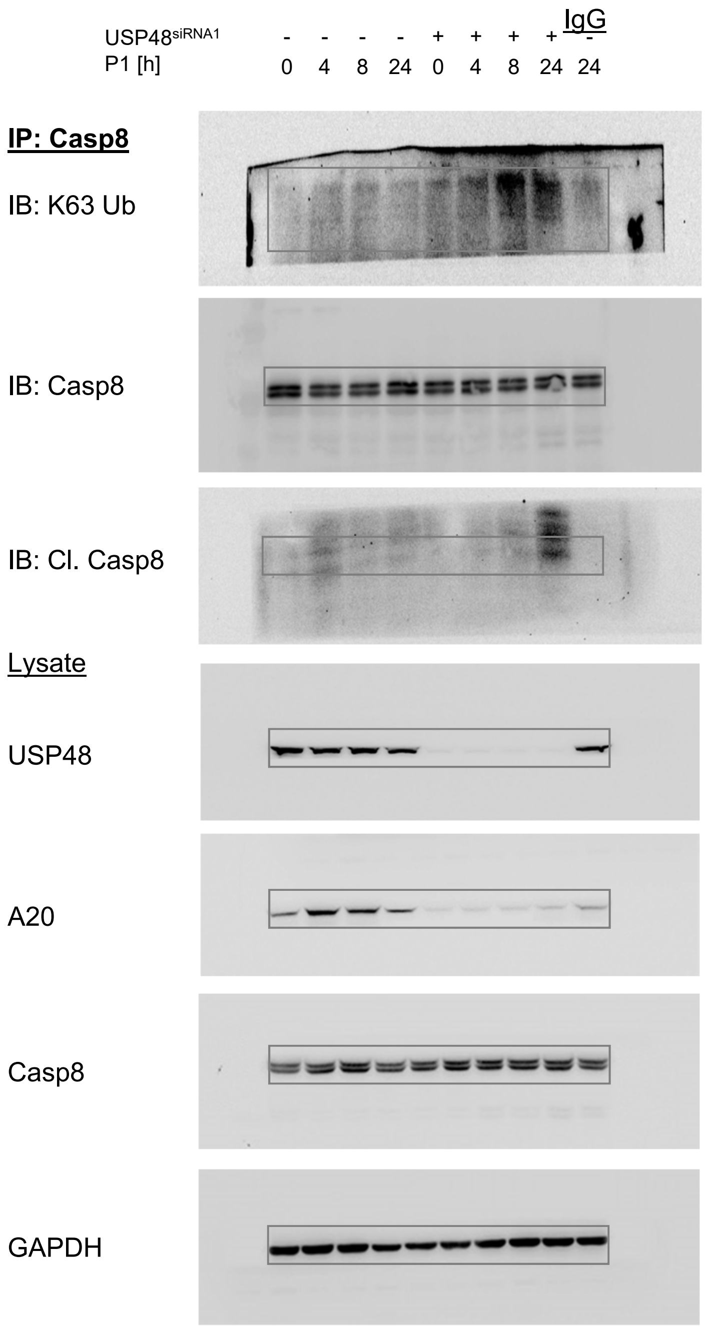

Uncropped blot for Fig 4a

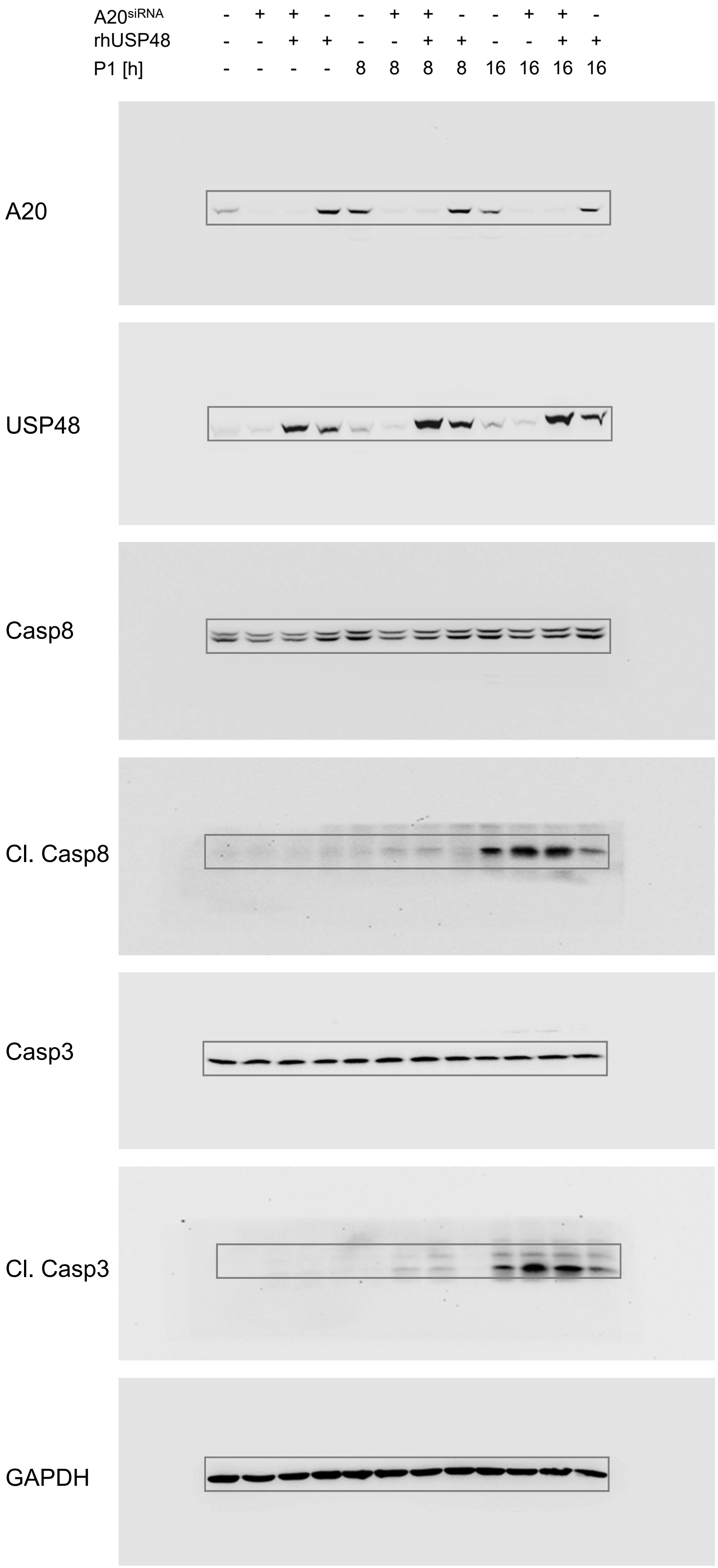

Uncropped blot for Fig 4c

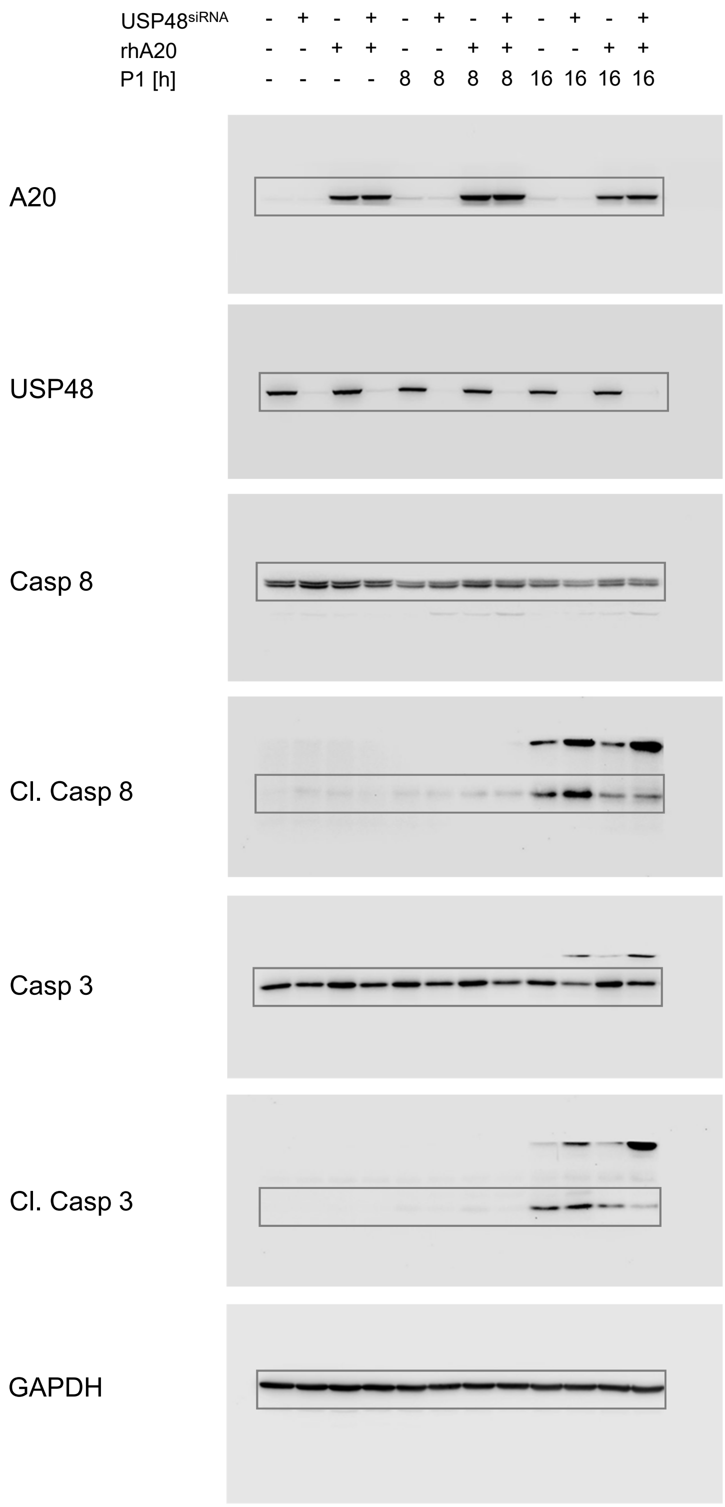

Uncropped blot for Fig S1a

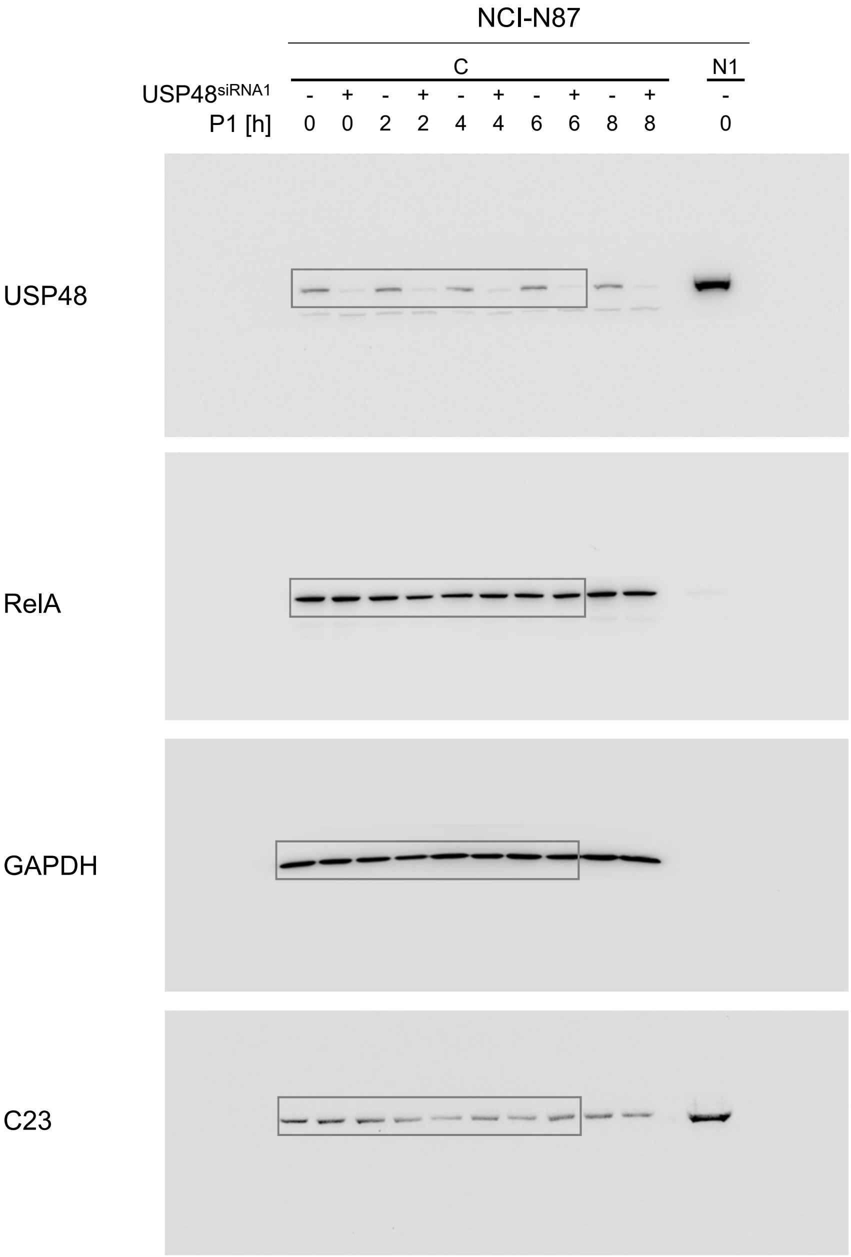

Uncropped blot for Fig S1a

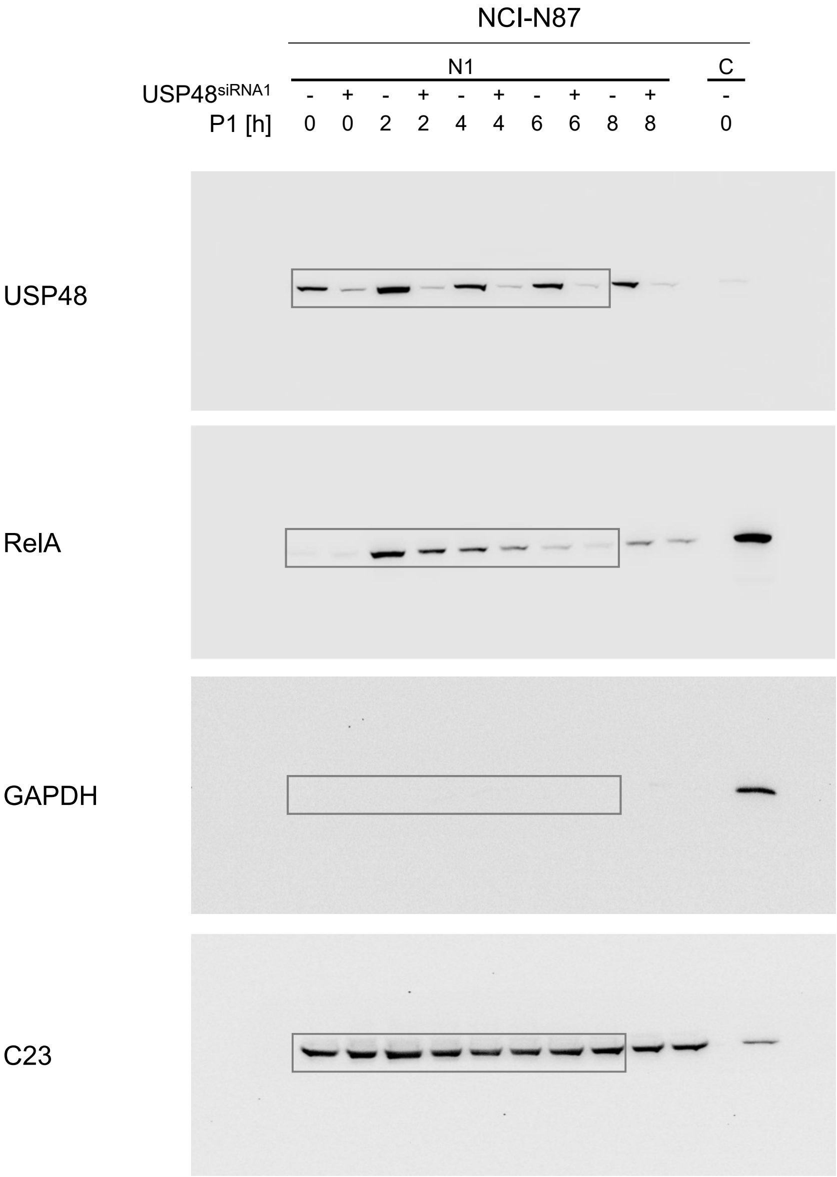

Uncropped blot for Fig S1b

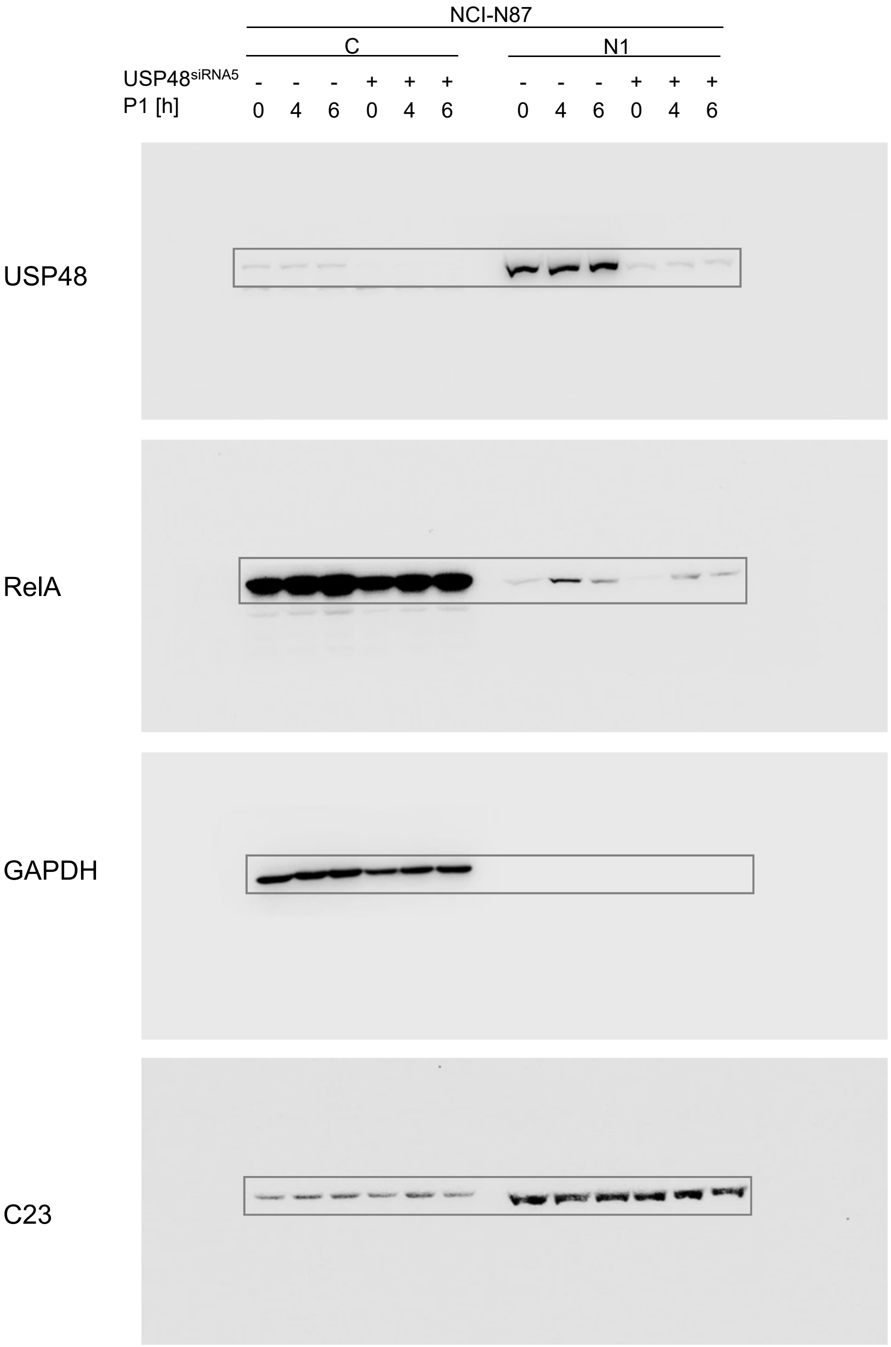

Uncropped blot for Fig S2

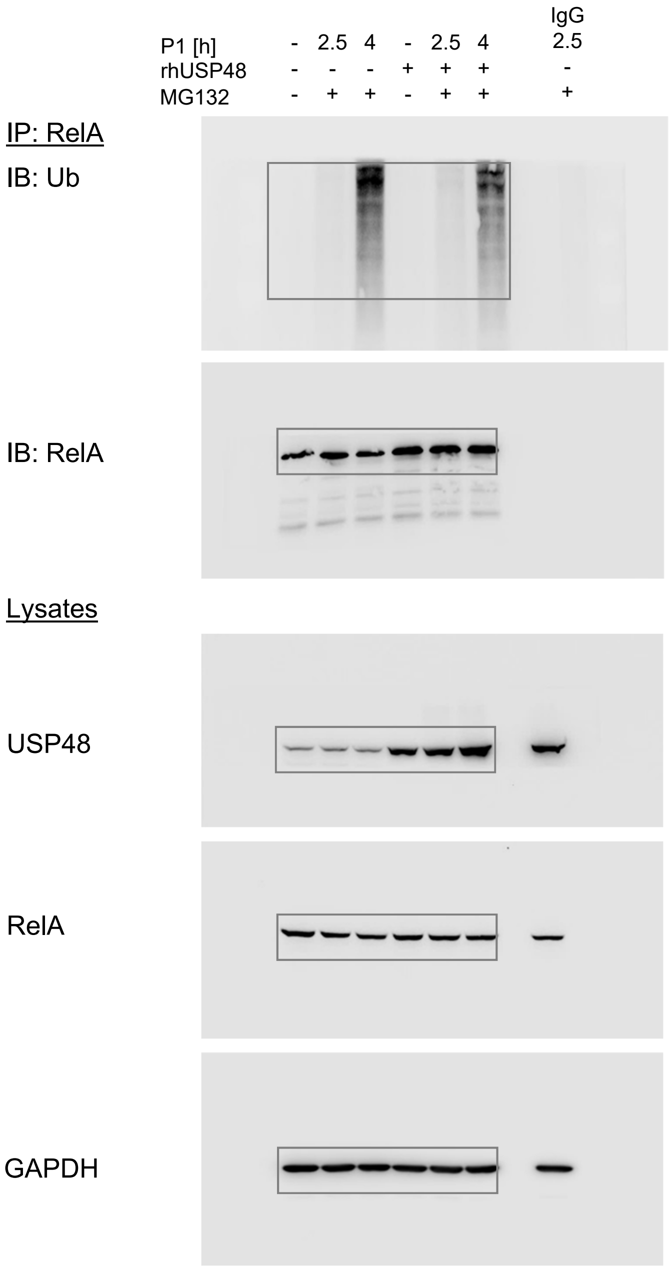

Uncropped blot for Fig S3a

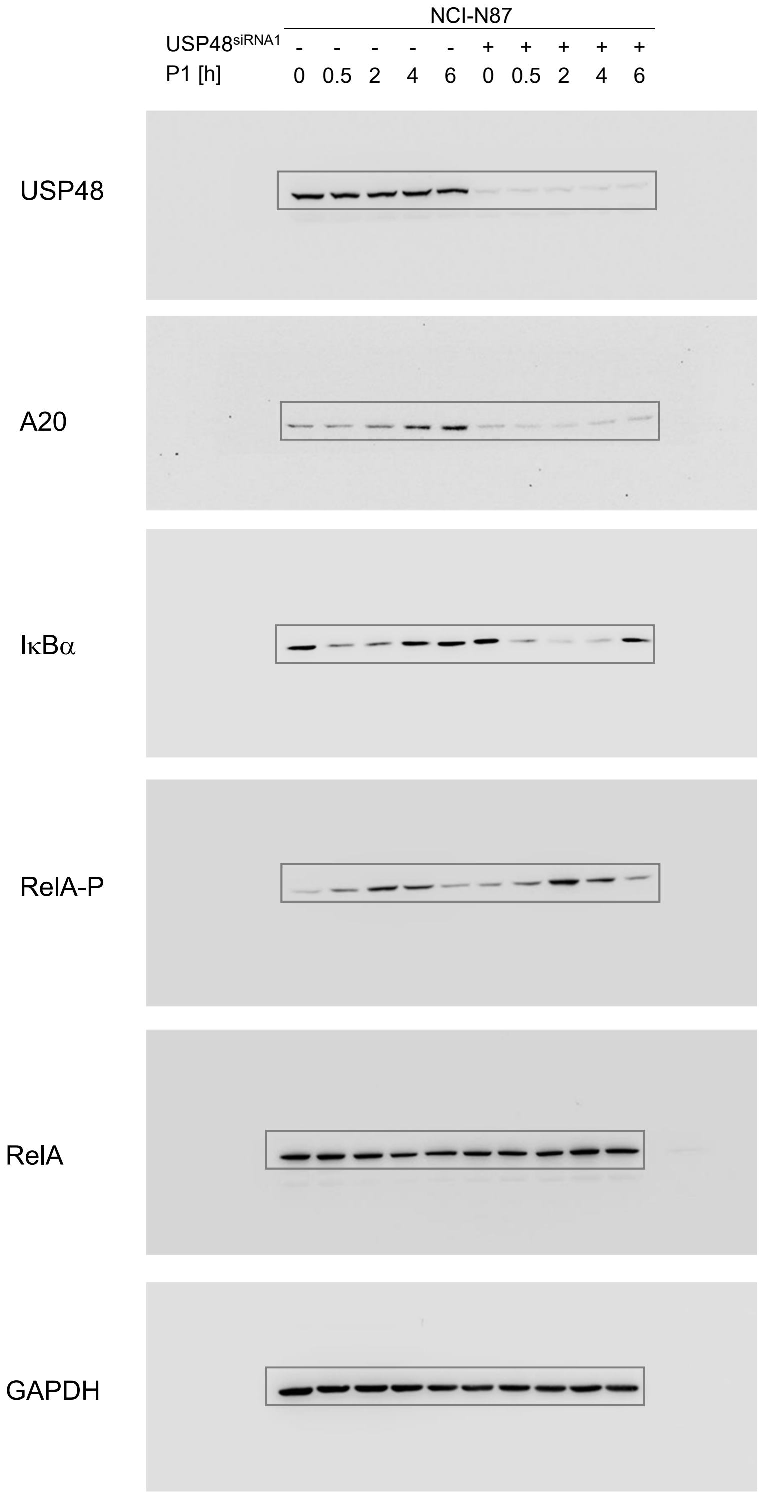

Uncropped blot for Fig S3b

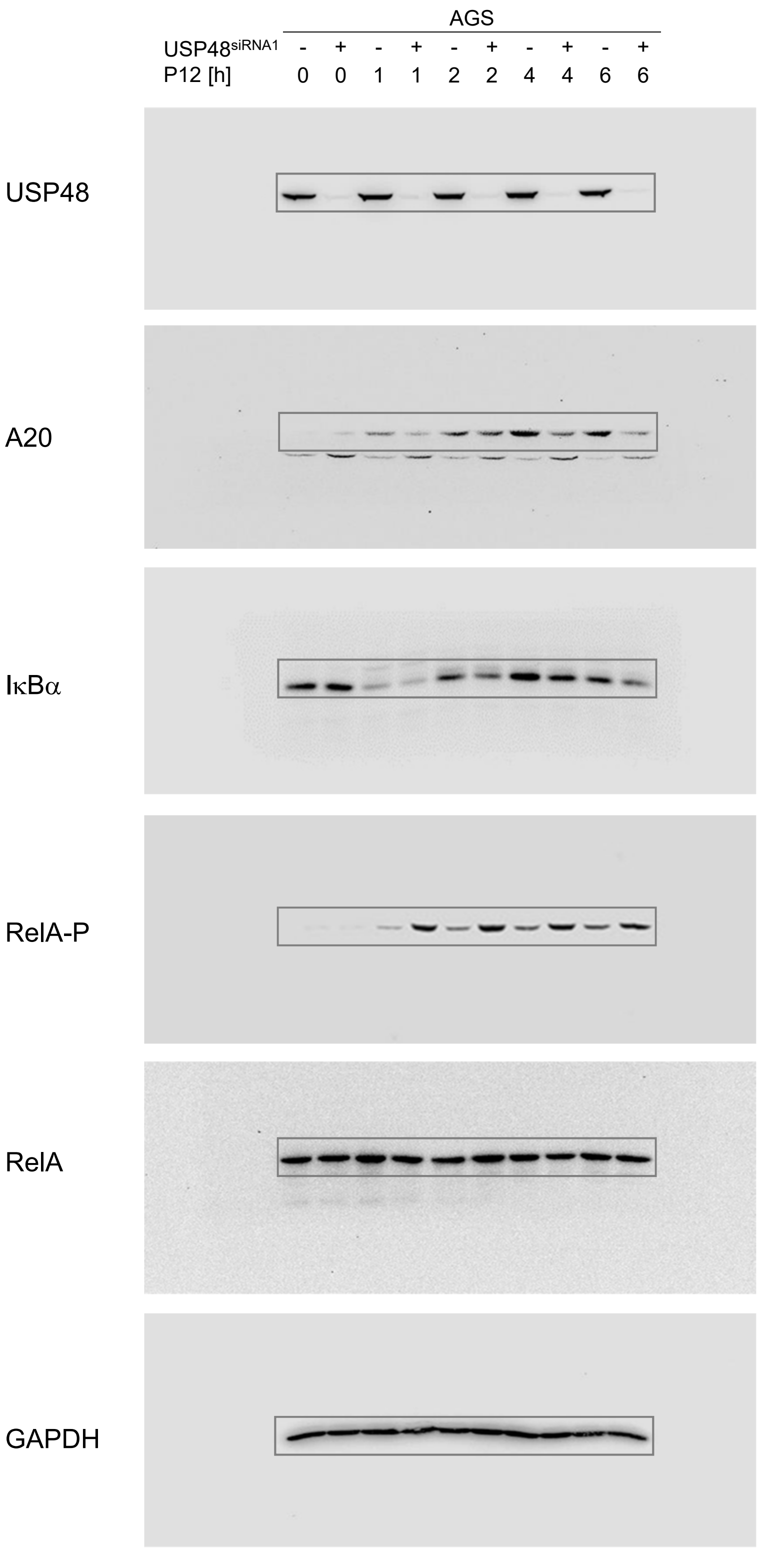

Uncropped blot for Fig S4a

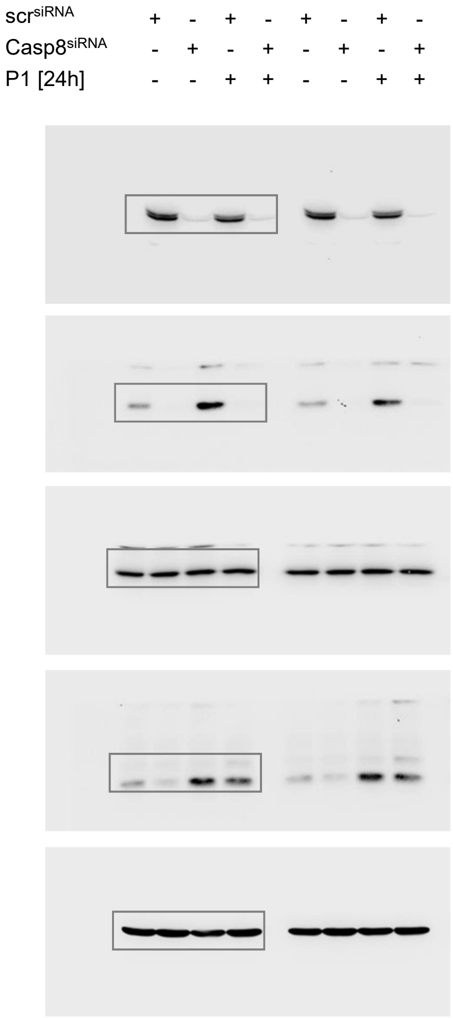

Supplement: Supplementary file 1 — Supplementary file1 (PDF 4288 KB) [file 18_2022_4489_MOESM1_ESM.pdf]
